# The Second Spiking Threshold: Dynamics of Laminar Network Spiking in the Visual Cortex

**DOI:** 10.3389/fnsys.2016.00065

**Published:** 2016-08-17

**Authors:** Lars E. Forsberg, Lars H. Bonde, Michael A. Harvey, Per E. Roland

**Affiliations:** ^1^Brain Research, Department of Neuroscience, Karolinska InstituteSolna, Sweden; ^2^Signalling Lab, Department of Neuroscience, Faculty of Health Sciences, University of CopenhagenDenmark

**Keywords:** dynamical systems, spontaneous activity, visual motion, object vision, evoked activity

## Abstract

Most neurons have a threshold separating the silent non-spiking state and the state of producing temporal sequences of spikes. But neurons *in vivo* also have a second threshold, found recently in granular layer neurons of the primary visual cortex, separating spontaneous ongoing spiking from visually evoked spiking driven by sharp transients. Here we examine whether this second threshold exists outside the granular layer and examine details of transitions between spiking states in ferrets exposed to moving objects. We found the second threshold, separating spiking states evoked by stationary and moving visual stimuli from the spontaneous ongoing spiking state, in all layers and zones of areas 17 and 18 indicating that the second threshold is a property of the network. Spontaneous and evoked spiking, thus can easily be distinguished. In addition, the trajectories of spontaneous ongoing states were slow, frequently changing direction. In single trials, sharp as well as smooth and slow transients transform the trajectories to be outward directed, fast and crossing the threshold to become evoked. Although the speeds of the evolution of the evoked states differ, the same domain of the state space is explored indicating uniformity of the evoked states. All evoked states return to the spontaneous evoked spiking state as in a typical mono-stable dynamical system. In single trials, neither the original spiking rates, nor the temporal evolution in state space could distinguish simple visual scenes.

## Introduction

Most neurons have a spiking threshold, above which they start to send out sequences of action potentials. This absolute spiking threshold separates two fundamental states of the neuron: below the threshold it only expresses fluctuations of the membrane potential; above the threshold it spikes and affects its post-synaptic neurons. Living brains have spontaneous ongoing spiking. In the visual cortex, there is spontaneous ongoing spiking when the eyes are closed as well as in absolute darkness (Hubel and Wiesel, 1959). When the eyes are open, the influx of action potentials from the retinae changes the spiking in the primary visual cortex (Hubel and Wiesel, 1959; Jung, 1959). Since then efforts have been made to separate the spiking evoked by external visual stimuli from the spontaneous ongoing spiking in single trials. Recently, Huys et al. (2016) analyzed the dynamics of spike trains and found a dynamical threshold that separated the spontaneous ongoing spiking from visual evoked spiking. The two forms of spiking were qualitatively different and separated by this second threshold, such that single trials could be separated into episodes of spontaneous spiking and visually evoked spiking in single neurons and small ensembles of neurons in layer 4 of the primary visual cortex. This, second spiking threshold is not apparent from traditional analyses of spike rates and spike timings or from statistical analysis of these variables. In addition Huys et al. (2016) showed that the spiking dynamics could be (mathematically) described as that of a mono-stable excitable system with one stable attractor surrounded by the second threshold. This implies, that for any external perturbation by a visual stimulus, the spiking will eventually return to the spontaneous spiking state. In this report, we examine whether the second threshold and the mono-stable dynamics are properties of the network in the primary visual areas 17 and 18 in the ferret. The purpose is not to compare the dynamical systems analysis with traditional spike train analyses as the two relate following the principle of complementarity: one gives a description of possible codes and information contained in the spiking, the other describes the dynamical rules that produce spontaneous and evoked spiking (Shenoy et al., 2013).

If the second threshold ofHuys et al. (2016) is a property of the network, it should transcend the scales with which the network is examined. Therefore, it should be present also in supra- and infra-granular layers, and be present also for the collective spiking of neurons across layers and present in all zones of the primary visual areas 17 and 18. The function of the second threshold is to keep the spontaneous spiking close to its fixed-point attractor and prevent that spontaneous spiking becomes evoked. We examined these predictions in different cortical layers and zones within areas 17 and 18. Similarly, if the network of neurons behaves as a mono-stable excitable system with one attractor, this should be the only attractor in state space, no matter where in areas 17 and 18 the electrode is placed. In Huys et al. (2016) the evoked spiking was driven entirely by an external, sharp visual transient. The sudden appearance of visual objects, drove the spiking across the dynamical threshold. We examined whether the network in areas 17 and 18 also behaves as a mono-stable network when it is continuous stimulated with moving objects in the field of view. Objects moving in the field of view give rise to both sharp and smooth visual transients. So, we examined whether smooth and gradually increasing visual transients can drive the spiking across the second threshold and into the evoked state. In Huys et al. (2016) the spiking returned to the spontaneous spiking state relatively quickly, so we examined whether the spiking can enter the evoked state several times. The second threshold separates state space into two domains: one for the spontaneous ongoing spiking and one for the evoked spiking. To understand the dynamics, we also examine the details in the spiking dynamics in single trials prior to and after the crossing of the threshold. The relevance of the answers to these experimental questions may be seen in the context of discover when and where in the network the neurons spike in the spontaneous ongoing state, how and how quickly the network can react to a visual stimulus, cross the second threshold and enter the evoked state driving the perception of the visual scenes. By examining the details of the evoked states, one might reveal whether the dynamics in the evoked states tells which of the different visual scenes the animal was exposed to.

We could confirm the observations by Huys et al. (2016). First, the network during spontaneous spiking and during visually evoked spiking behaved as a mono-stable excitable network. Second, the two forms of spiking were qualitatively different and spontaneous ongoing spiking and visually evoked spiking evolved in two adjacent domains of state space, separated by the second threshold (that in mathematical terms is a separatrix). This implies that, dynamically it is easy to discriminate single trials being evoked from single trials remaining in the spontaneous ongoing state. We found that the second threshold was present in all cortical layers and zones and also present in simultaneously recorded neurons across all layers, indicating that it is dynamic property of the network. Dynamically, in single trials, the spiking is either in the spontaneous state or in the evoked state. However, during a single trial, the spiking can return and enter the evoked state several times. Logically, only when in the evoked state, the spiking dynamics should reveal which visual scene is presented. Our results, however, showed, that although the temporal dynamics preserved information about the visual transient (sharp or smooth), neither the state space *temporal* dynamic behavior in single trials, nor the single trial instantaneous spiking rates were able to distinguish all simple visual scenes to which the animals were exposed.

## Materials and methods

### Animals

All experimental procedures were approved by the Stockholm Regional Ethics Committee and were performed according to European Community guidelines for the care and use of animals in scientific experiments. Recordings were performed in 13 adult, female ferrets, of which 8 also participated in the Harvey and Roland (2013) study. Ferrets were initially anesthetized with Ketamin (15 mg kg^−1^) and Medetomidine (0.3 mg kg^−1^) supplemented with Atropine (0.15 mg kg^−1^). After the initial anesthesia ferrets received a tracheotomy and were ventilated with 1:1 N_2_0:0_2_ and 1% Isoflurane. The arterial pCO_2_ (partial pressure of CO_2_) was maintained between 3.5 and 4.3 KPa. A craniotomy was made exposing the left hemisphere visual areas 17, 18, 19, and 21 and was covered with a chamber affixed to the skull with dental acrylic. Animals were paralyzed with pancuronium bromide (0.6 mg kg^−1^), the left eye was occluded, and the right eye had its pupil dilated (1% atropine), nictating membrane retracted (10% Phenylephrine), and was then fitted with a zero power contact lens.

### Stimulation

**Figure 1:**
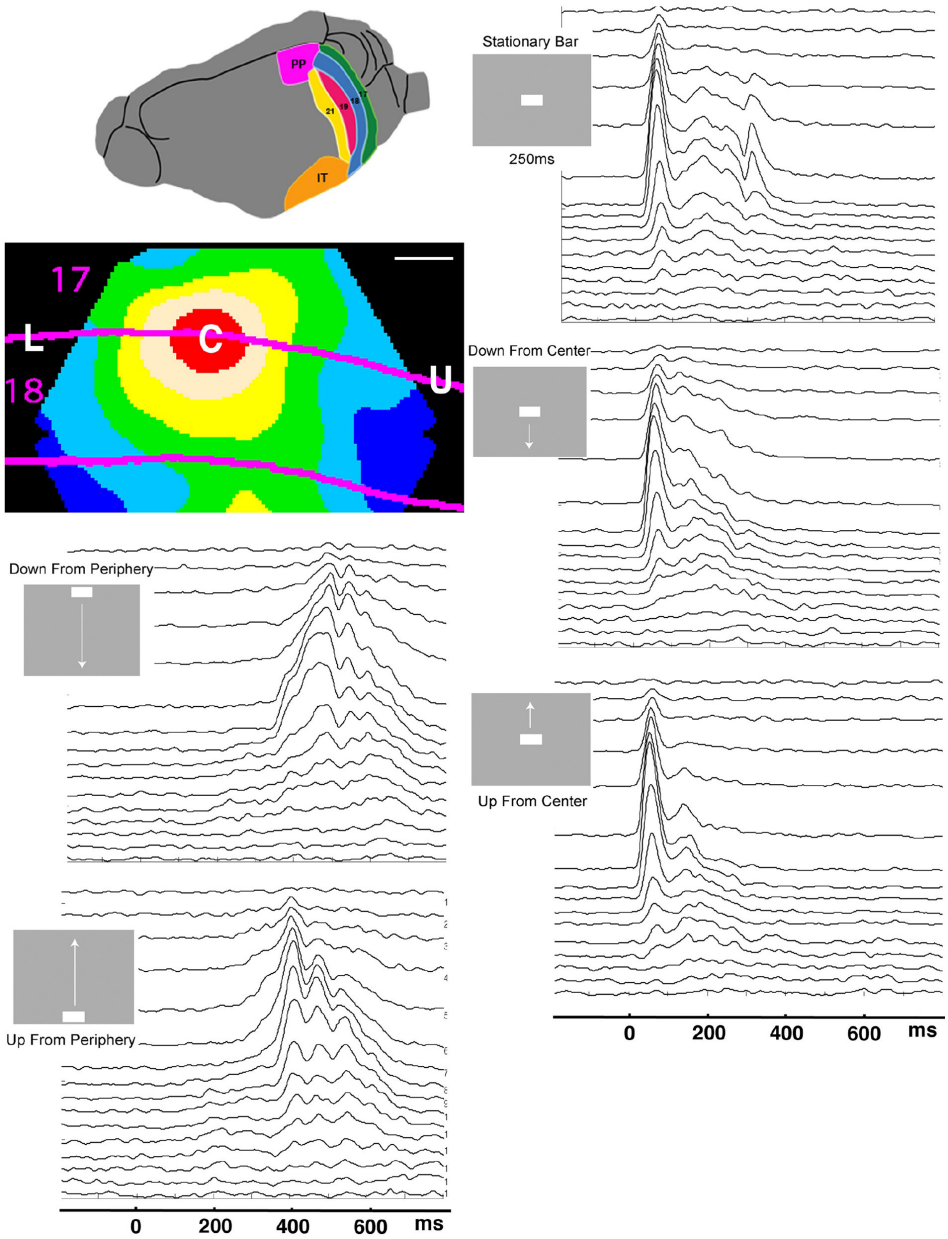
**Laminar post-stimulus-histograms of the multiunit spiking to a single moving bar**. Top left: posterior visual areas of the ferret. PP, posterior parietal; IT, inferior temporal. Below: Mean voltage sensitive dye signal of 7 animals 100 ms after appearance of a stationary bar at the CFOV. Red: C, zone mapping bar at CFOV; beige: edge zone; The lower part of the visual field is mapped between L and C, the upper visual field between C and U. Scale bar: 500 μm. Time scale 0 = start of stimulus. The smoothed (σ = 10 ms) spike rates from all 16 leads in all 5 stimulus conditions have identical time scales. The layer 1 lead recordings are in the top of the panels.

After determining the receptive field (RF) with the m-sequence method, and a single tungsten electrode (Reid et al., 1997) at the estimated position of the vertical meridian at the area 17/18 border, the position of the visual display screen was adjusted so as to be precisely centered to this RF location (Harvey and Roland, 2013). Stimuli were presented in a pseudorandom order on a video monitor with a refresh rate of 120 Hz located 57 cm in front of the animal. Stimuli were controlled using a VSG series IV system (Cambridge Research Systems, Kent UK). Stimuli consisted of 1 × 2° horizontal bars (64.5 cd m^−2^) on a homogenous gray background (7.2 cd m^−2^). There were six conditions: (1) A bar moving downwards from the center of field of view (CFOV) with a velocity of 25.4° s^−1^ for 413 ms along the vertical meridian. (2) a bar moving upward from the CFOV with a velocity of 25.4° s^−1^ for 413 ms along the vertical meridian. (3) upward and (4) downward moving bars initially flashed 10.5° below and 10.5° above the center of field of view (CFOV) respectively and moving a total of 21° with a velocity of 25.4° s^−1^ along the vertical meridian for a period of 825 ms with start and end points equidistant from the screens center (Figure 1). (5) A single stationary bar was presented at the CFOV for 250 ms. In addition (6) a no-stimulus condition was included, consisting of a homogenous gray screen (7.2 cd m^−2^) presented continuously in between the stimulus conditions. Each of these seven conditions was repeated 50 times.

### Electrophysiology

We made 82 electrode penetrations perpendicular to the cortical surface along the estimated course of the vertical meridian using single shank, 16 channel, laminar probes (NeuroNexus, Ann Arbor, MI) with recording site resistances of 2–3 MΩ, and leads separated by 100 μm. For multiunit recordings the signal was digitally band pass filtered between100 Hz–10 KHz. and for local field potential recordings between 1 Hz and 10 KHz. All subsequent analysis was done using Matlab R13 (The MathWorks, Natick, MA). Spike detection was done off-line at 3 standard deviations from the mean noise level. At each recording site, receptive fields were first mapped using the methodology noted above. For electrode penetrations in cortex mapping the CFOV, the receptive fields in the granular layer were less than 3°.

### Cytorarchitectonics and functional retinotopy

**Figure 2:**
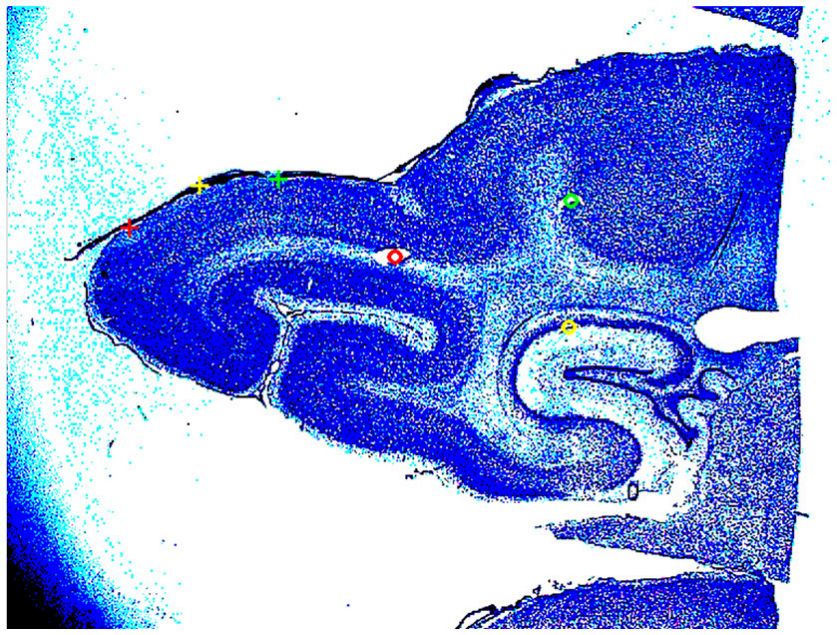
**Nissl stained section of the posterior part of the brain of animal 4**. The two yellow circles and the red circle show the cannula made holes used for the alignment of the slices. The red, yellow, and green cross, mark the borders between areas 17/18, 18/19, and 19/21. The detailed cytoarchitecture should be seen in higher resolution. The slices were reconstructed to a volume with the areal borders on the surface.

At the end of the experiment, three vertical needle marks were made around the recorded area, the animals were sacrificed (pentobarbital) and perfused trans-cardially with 4% paraformaldehyde. Brains were sectioned and alternate 50 μm sections were stained for Nissl and cytochrome oxidase. Areal borders were then reconstructed using cytoarchitectonic landmarks (Figure 2), and these borders were mapped onto the image of the cortical surface in each animal containing the positions of all electrode penetrations. After reconstruction, the cytoarchitectural borders of individual animals were aligned by simultaneous standard affine transformations as described in Harvey et al. (2009). In this way, also the electrode penetrations could be mapped on a common map.

**Figure 3:**
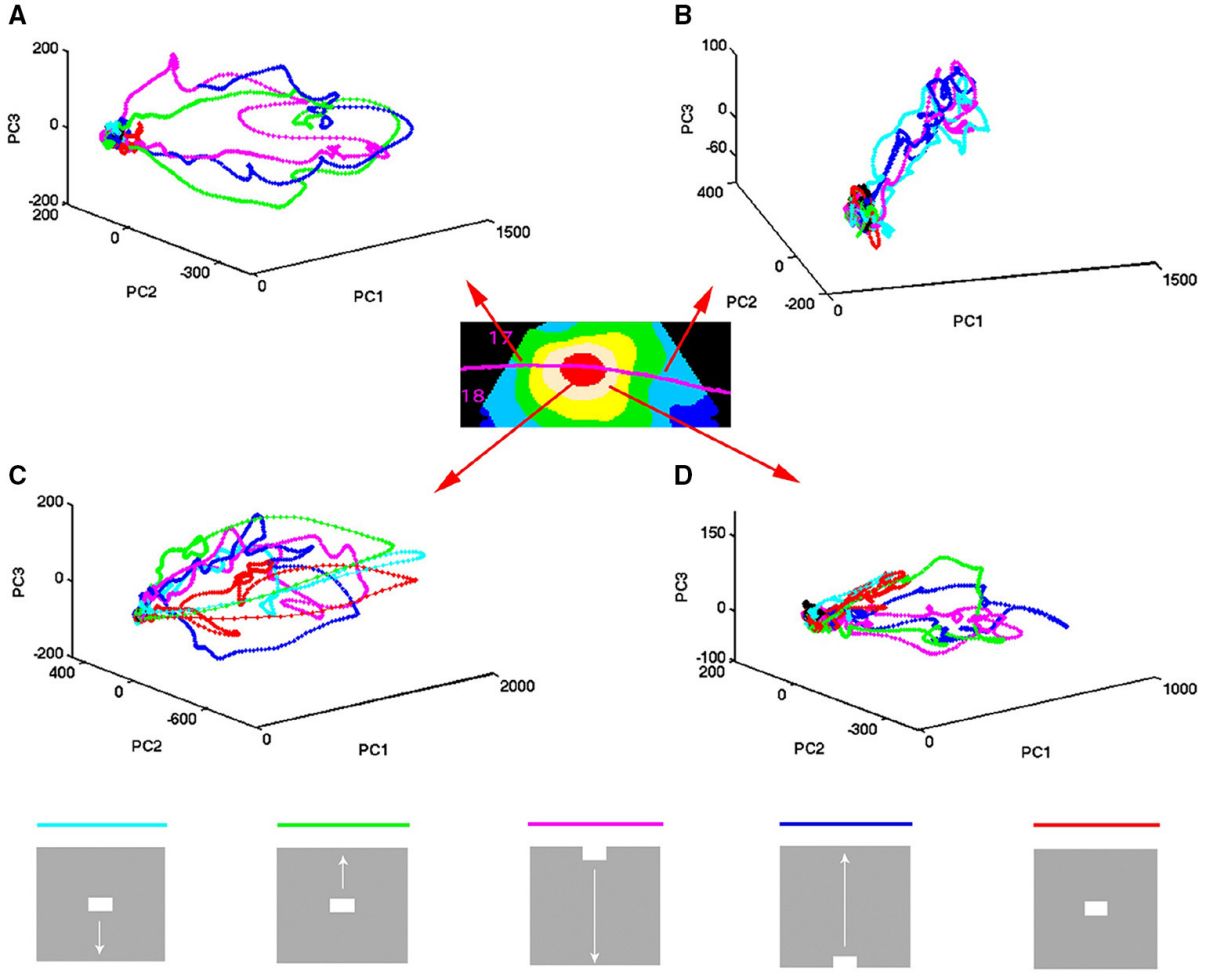
**Temporal spiking dynamics**. The color coded trajectories in 3-dimensional state space made by the axes of the first 3 principal components show the spiking dynamics of each condition (Materials and Methods) for the 4 electrode penetrations shown, one from each of the 4 cortical zones shown in the insert. **(A)** Maps the bar moving down from the peripheral FOV (early) and the bar moving up from the peripheral FOV (late), plus the bar moving up from the CFOV. **(B)** Maps the bar moving up from the peripheral FOV (early) and the bar moving down from the peripheral FOV (late), plus the bar moving down from the CFOV. **(C)** Maps all bars in or passing through the CFOV. **(D)** Maps all moving bars (The stationary bar, flashed in the CFOV produce a minor initial spiking here). The trajectories are composed of points, one for each ms, from which the speed by which the dynamics evolves can be appreciated. Note the chaotic-like evolution of the trajectories of all conditions in the pre-stimulus period and how the speed increases when the stimulus transients influence dynamics. The first 3 PCAs accounted for 64–83% of the variance. The values on the axes are in units of the principal components. The fixed point is located at (0,0,0).

The electrode penetrations were all located in area 17 and 18. Six of the animals also had a measurement of voltage sensitive dye signal to the stationary 1 × 2° bar. The red zone in Figure 1 marks the 90% increase to this bar of these animals when the cortex was aligned. The electrode penetrations were further subdivided into (1) 17 penetrations within the cortical space mapping the bar at the CFOV (Figure 1, red zone); (2) 16 edge penetrations, (beige zone, Figure 1); (3) 20 penetrations roughly along the lateral part of the border between the areas 17 and 18 (up to a distance of 600 μm) outside (1) and (2); (4) 29 penetrations roughly along the medial border between areas 17 and 18.

### Construction of Figure 1

Seven of the animals also had a voltage sensitive dye measurement of the changes in the membrane potentials associated with the sudden exposure to the stationary bar. After alignment of the cytoarchitectural borders and similar alignment of the voltage sensitive dye measurements a population map was made showing the average voltage sensitive dye signal changes induced by the presentation of the. Figure 1 is a snapshot of this population movie at the time when the signal in the red zone was maximal.

### Data analysis

For a given electrode position, we examined the inter spike time intervals in multi-unit recordings in all 50 trials. Given spike n in a spike train from one trial, we examined the time from spike n−1 to n and plotted that against the time from spike n to spike n+1. All pairs were plotted in all trials, condition by condition. The purpose was to examine any periodicity or gamma oscillations in the data. Second, to see if the instantaneous rates of evoked trials differed from other trials. The plots in Figure 3A show the color-coded density of points in an area of 0.5 × 0.5 ms.

### Current source density

We estimated current source densities from the second spatial derivative of the local field potentials in filtered (0.5–600 Hz) original data files (Rappelsberger et al., 1981). The data files were averaged over 50 trials for each lead either from a single stationary bar or from whole screen flashes 1 of 1 Hz. Layer 4 was estimated to start where the early sink appeared (the sink being at the upper part of layer 4), the supragranular and infragranular layers above respective below layer 4 (Supplementary Figure 1; see also Harvey et al., 2009).

### State-space analysis of spiking

For each animal, penetration, condition, and trial, the spike time histograms (in 1 ms bins) were smoothed by a Gaussian kernel, window 15 ms; σ = 6 ms. This gave a time series of 1000 observations of the spike rate. There were 50 trials per condition and 6 different conditions giving a total of 300 trials per electrode penetration. As each trial had 1000 observations this gave a total of 300,000 observations per electrode penetration.

PCA of the instantaneous spike time histograms was made to reduce the dimensionality of leads. PCA was performed, using Singular Value Decomposition as implemented in the Matlab PCA routine.

$$X\;=\;U\Sigma V^{T},X\in {\mathbb{R}}^{N\times L},U\in {\mathbb{R}}^{N\times N},\Sigma \in {\mathbb{R}}^{N\times L},V^{T}\in {\mathbb{R}}^{L\times L}$$

where *N* = *N*_*times*_·*N*_*trials*_·*N*_*conditions*_ = 1000·50·6 = 300 000 is the number of observations and *L* = 16 is the number of leads.

The vectors **U** = [***u*_1_*u*_2_*u*_3_…*u_N_***] hold the coordinates of the observations in the PCA vector space spanned by **V^T^** = [***v*_1_*v*_2_*v*_3_…*v_L_***]^**T**^. The diagonal matrix $\Sigma \;=\;\left[\begin{array}{ccc} \sigma_{1} & 0 & 0\\ \ddots \\ 0 & 0 & \sigma_{L} \end{array}\right]$ holds the singular values which scales vectors in **U** to reflect the variance explained by each of the *L* principal components.

Two types of PCA were made, one on all 16 leads (16 columns) and one for the 3–4 leads in each layer (3 columns in granular layer and 3 columns in supragranular layer and 4 columns in the infragranular layer).

First, the data were centered. First we calculated the mean of each column. Then we subtracted this mean from each of the elements in that column. This operation transforms the spike rates to the new variable **X** (rate fluctuations from the mean of the 16 leads). Then the data were projected into their new coordinate system, i.e., the space spanned by the principal component axes via PCA as described above. The coordinates in the PCA space are the elements in **U**. In the PCA vector space, the i'th observation (i'th row in **X**, [***x*_*i*1_*x*_*i*2_*x*_*i*3_…*x_iN_***]) is located in the coordinate (***u*_1*i*_**σ_1_, ***u*_2*i*_**σ_2_, ***u*_3*i*_**σ_3_) where ***u*_1*i*_** is the i'th element in the vector ***u*_1_**. As seen from the equation above, it is possible to reconstruct the i'th row of **X** (with mean centered columns) by the following calculation:

$$\left[x_{i1}x_{i2}x_{i3}\ldots x_{iN}\right]\approx u_{1i}\sigma_{1}{v_{1i}}^{T}+u_{2i}\sigma_{2}{v_{2i}}^{T}+u_{3i}\sigma_{3}{v_{3i}}^{T}$$

The state-space trajectories in 3 dimensions are the ***(u*_1*i*_, u_2*i*_, u_3*i*_)** coordinates, plotted over a time series of i's (Figures 2, 3). Note that the (***u*_1*i*_, *u*_2*i*_, *u*_3*i*_**)≠ (***x*_*i*1_*x*_*i*2_*x*_*i*3._**). Figure 2 shows the trajectories of the first 3 components. Figure 3B shows a single trial projected in 3-dimensional state space viewed from different sides. The % of total variance accounted for by the first 3 components was 67.3.

### Testing differences in trajectories

The original spike data were binned in 10 ms bins. A PCA was done separately for the granular, supragranular and infragranular layers for each electrode penetration and the trajectories for 2 different stimulus conditions were plotted in 3-dimensional state space. For each 10 ms the Euclidian distances between the two trajectories in the 3-dimensional state space were calculated. This was repeated for each of the penetrations located in the same cortical zone. Then the differences for each 10 ms time segment were tested under the H0 of no difference with a false discovery rate of 0.05 with correction for family wise error (Benjamini and Hochberg, 1995).

### Estimating the separatrix

**Figure 4:**
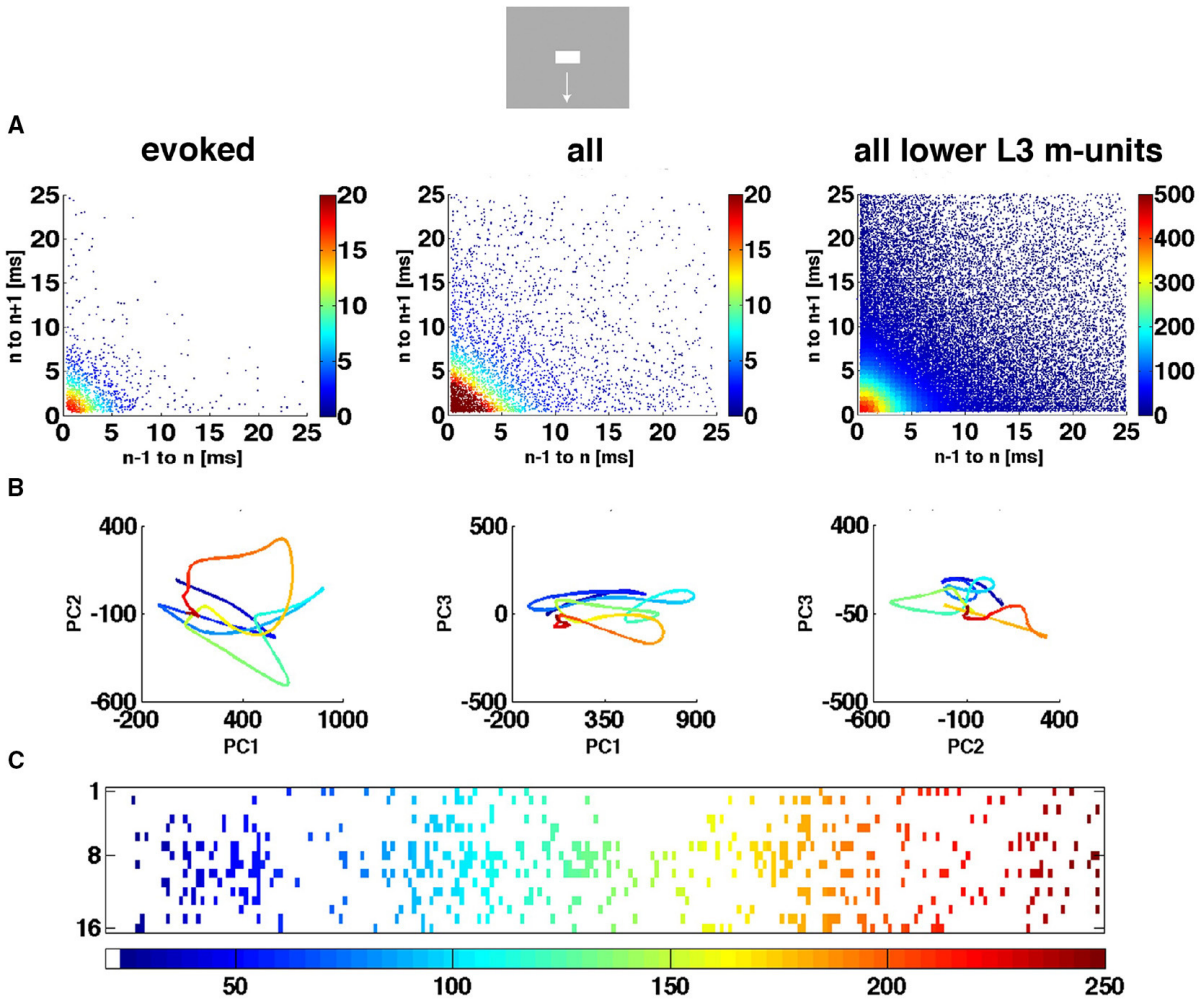
**The spiking dynamics reflect the collective spiking in all cortical layers**. **(A)** Plot of inter-spike intervals of lower layer 3 multi-units located in the CFOV mapping zone. Left: inter-spike intervals (*n* = 1263), from evoked trials only, in ms from a single electrode penetration in response to a bar moving down from the CFOV. Middle: same electrode penetration inter-trial intervals (*n* = 5207) for all trials for the bar moving down from the CFOV. Right: inter-spike intervals (*n* = 30.774) for all lower layer 3 leads in the cortex mapping the CFOV. Note the absence of any periodicity. The color scales show the number of observations within 0.5 × 0.5 ms space. **(B)** Trajectory of a single trial (trial 30), from 0 to 250 ms in 3 different projections of state space generated by PCA color coded in time after stimulus to compare with **(C)**. First 3 components accounted for 77% of total variance. **(C)** Same trial as **(B)**, all spikes from all leads. All figures, except **(B)** left from animal 6, electrode penetration 2.

Velocity vectors were calculated as the temporal derivative of the single trial trajectory in 1 ms steps at successive positions. These velocity vectors are shown in Figure 4 as one arrow per ms.

The velocity vectors describe the flow field in two dimensions (Figure 4). In the control condition (gray screen) these single-trial velocity vectors can point in all directions. For trajectories taking a course away from (0,0), the velocity vectors point away from (0,0). When the trajectory reach a certain distance from (0,0) the velocity vectors bend off and point toward (0,0) (Figure 4A). After a stimulus appear, the vectors in some stimulus trials may bend off as did the vectors in spontaneous ongoing spiking, but the majority will continue escaping from (0,0) pointing away from the fixed-point at (0,0) (Figure 4B). There is thus a border at a certain distance from the fixed point with diverging vector flows in state space. Mathematically, this border with diverging vector flows on either side is a separatrix.

We estimated the position of this separatrix by adjusting the position of a rectangular box to a position separating trials with vectors bending off and pointing again to (0,0) from trials with vectors pointing outwards and away from (0,0). A practical solution was to minimize the number of trajectory points (one per 1 ms) outside the box of trajectories bending off and pointing toward the (0,0). In addition the number of points of trajectories with vectors pointing away from the box should be maximized. We used a practical solution to this problem by setting a false discovery rate of 0.001 (Benjamini and Hochberg, 1995) that any bending trajectory point at any time was outside the box. In practice this corresponded well to the position of the diverging vector flows (Figure 4).

Plotting all single trials velocity vectors in the 2D state space spanned by PC1 and PC2 just give a uniform blue or red clot. Therefore, we used the Matlab® randperm routine. The order of all 50 trials was randomly permuted such that trials from different electrode penetrations and animals now became neighbors. We then selected a small number (1,2,10) of the first such permuted sequence of trials and plotted them as in Figure 4C to better illustrate the single trial vectors.

#### The fate of the stuck trials and evoked trials

From the estimate of the position of the separatrix, we separated the single MUA spiking trials into spontaneous ongoing spiking trials or stuck trials staying within the separatrix in the post stimulus period and evoked trials escaping the state space demarcating the separatrix in the post stimulus period. An evoked trial thus consisted of episodes of time when the spiking was in the evoked state. Then we calculated the proportion of evoked trials for electrode penetrations in each of the 4 cortical zones.

### Conditional proportion evoked

First PCA was calculated separately for 3 leads covering the supragranular layers, 3 leads covering the granular layer, and 4 leads covering the infragranular layers. Then trials were sorted in episodes of being evoked and episodes of being stuck (inside the space bordered by the separatrix). Thus for a certain ms, a trial from a particular stimulus condition is either in the evoked state or stuck. Given that a trial is evoked in the granular layer, is that trial also evoked in the same ms in the supragranular layer? By counting such conditional events from all electrode penetrations covering the CFOV zone, we got a conditional probability estimate for each ms that a trial would be evoked.

Similarly, given that the trial was stuck in the granular layer at a certain ms, we examined whether the trial was evoked in the supragranular layers. We counted then all such events from all electrode penetrations covering the CFOV zone and plotted these conditional probability estimates in **Figure 9**.

## Results

### Cortical mapping of the moving objects in the center of field of view

For most of the time spent in the experiments, the ferrets were at rest, exposed to a uniform gray screen in an otherwise totally dark room. In experiments, the uniform gray visual screen was suddenly changed by the appearance of a moving bar. When the bar was flashed directly in the center of field of view, CFOV, the voltage sensitive dye signal increased to reach maximum after 100 ms in the part of the cortical network of neurons labeled C in Figure 1. The red C zone is located on both sides of the cytoarchitectural border separating areas 17 and 18.

When a moving bar appeared in the CFOV, this change in the field of view, FOV, excited a population of neurons at rest in layer 4 to produce a fast increase in the number of action potentials: a classical, sharp, transient ON response, an ON r(t). As the change in the visual field was equal to the appearance of the bar, one may say that the bar was initially mapped by the ON r(t) in the central C-zone. Here the ON r(t) transient spread to the remaining cortical layers (Figure 1). New neuron populations mapped the moving bars successively, because the eyes were paralyzed (Materials and Methods). Consequently the spiking intensity decreased in the red C-zone as the mapping of the bar progressed into the adjacent beige edge zone of the cortical network. Table 1 shows the epochs during which the spiking, r(t), was significantly increased compared to the rest condition for the neurons in the different cortical layers all mapping the CFOV (False discovery rate, *p* < 0.05, corrected for family-wise error, *n* = 17 electrode penetrations). Note that in conditions where the bar appeared in the peripheral FOV, there is a delay of 200 to 300 ms before the multi-units mapping the CFOV become significantly active (Table 1).

**Table 1:** **Epochs of increased spiking in ms from the start of stimulus in CFOV mapping neurons**.

**Condition**	**Supragranular**		**Granular**	**Infragranular**
Stationary bar	40–230		50–410	n.s.
Bar moving down from CFOV	30–60	160–240	30–370	100–280
Bar moving up from CFOV	20–330		30–320^*^	50–370
Bar moving down	300–530		300–500^*^	190–730
Bar moving up	400–710^*^		260–750	280–750

*All epochs significant at p < 0.05*,

* *p < 0.01 (see Section Materials and Methods)*;

*n.s. not significant*.

Figure 1 also contains the rates induced from exposing the ferrets to a stationary bar for 250 ms, for comparison. The most noticeable difference is that the instantaneous spiking rates from the network mapping the CFOV for the stationary bar had an OFF response. This was in contrast to the four examples of appearances of moving bars. The stationary bar disappeared from the FOV, whereas the moving bars continued to move in the FOV and then subsequently got mapped by adjacent cortical populations, because the eyes were still (Materials and Methods). The moving stimuli that were introduced in the peripheral FOV also had an initial ON r(t) in the cortex mapping the peripheral FOV (data not shown; see Harvey et al., 2009).

This traditional analysis of spiking rates does not tell anything about the spiking dynamics (Brette, 2015). There is no obvious threshold separating spiking rates in the pre-stimulus period from rates in the post-stimulus periods. To examine whether there is a dynamical threshold in supragranular layers and infragranular layers one must do an analysis of the spiking dynamics. Figure 1 indicates that the cortical visual network can react to moving objects in two different ways: by sharp transients and by smoothly increasing rates. It is not known whether this smooth type of spiking dynamics also has a dynamical threshold. Neither is the dynamics known when the network of neurons is exposed to moving stimuli, i.e., whether spiking is under influence of one or more attractors. In order to investigate these questions, we mathematically analyzed the dynamics of the spiking in the cortical network of areas 17 and 18. In the remaining part of the Results section, we examine the cooperate spiking from 3, 4, or 16 leads of each electrode penetration.

### Evolution of spiking in state space

The spiking data went through a principal component analysis (PCA). The state space is multi-dimensional with the number dimensions equal to the number of principal components. In this state space, the spiking of all simultaneously recorded neurons at one ms is represented as one point in state space, corresponding to one state of these neurons. By mapping the evolution of states for the neurons sampled by all 16 leads of an electrode penetration, one can follow the collective evolution of the spiking states of these neurons as one trajectory in state space. This is done in Figure 3 showing the 3-dimensional state space made by the first 3 principal components (Materials and Methods). As the evolution in time is marked for each ms, the reader can compare with the post-stimulus histograms of the rates and with spike timing rasters (Figure 3 and Supplementary Figure 2) However, one should remember that each trajectory in state space show the cooperate spiking of all 16 multi-units.

At first glance there are two qualitatively different trajectories. Some trajectories evolve rather slowly, unpredictably, around (0,0,0), changing directions often. Other trajectories start in the space occupied by the former trajectories and escape to explore large parts of state space (Figure 3). In the cortical zones that do not map the object, the trajectories are of the first type [slow, chaotic-like and restricted to a small surround of (0,0,0)]. In the cortical zones mapping the moving bar, the trajectories escaped and explored large parts of state space (Figure 3). The trajectories from other electrodes in the 4 respective zones behaved similarly to the 4 examples shown.

A closer look at Figure 3 shows that the trajectories can escape either with fast initial speed that eventually slows down after which the trajectory changes direction and slowly returns to the zone around (0,0,0). This type of trajectory was associated with spiking dynamics driven by a sharp transient, i.e., the sudden appearance of the bar (examples red, light blue and green trajectories in Figure 3C). When the dynamics was driven by smooth transients such as a bar moving to be retinotopically mapped by the population of neurons mapping that part of the FOV, the trajectory speed slowly increased toward a maximum for thereafter to decrease. Then the trajectory returned to the zone near (0,0,0) (e.g., magenta and dark blue trajectories in Figures 3A,C).

We did not observe limit cycles, i.e., dynamics suggesting periodic oscillating spike dynamics. We examined the inter spike intervals in the electrode leads and plotted the dependence of any two spike intervals adjacent in time. That is, we plotted spike interval n−1 to n vs. spike interval n to n+1 (Figure 4A). The plots of these multi-unit spike intervals did not show any preference for location near the diagonal (*n* = 1312 electrode leads), meaning that we found no evidence of periodic spiking dynamics. In these plots an abundance of spiking in the gamma range 40–100 Hz would show up in the area outside the 10 ms x- and y- coordinates. The data did not support the existence of gamma spiking activity as this part of the plotting area had the least density of observations (*n* = 1312) as seen in the typical examples in Figure 4A.

Figure 4B shows the evolution of the spiking dynamics in a single trial of the downward bar moving condition. Figure 4C shows all spikes from all 16 leads for the same trial. Lead 7 has a high number of spikes of which the inter-spike intervals are shown in Figure 4A. Figure 4 illustrates that the single trial trajectory, neither relate to the instantaneous spiking rate, nor to the spiking rates of the fastest spiking leads. Calculated across all 16 leads, the trajectory reflects the collective spiking simultaneously recorded from all cortical layers by all electrode leads. In addition, the spiking trajectory can leave the surround of (0,0) and return, just to escape again several times. However, as a typical dynamical system, the trajectory never repeats itself. Additional examples of single trial trajectories and spike raster from the same trail for other conditions and the 4 cortical zones are shown in Supplementary Figure 2.

Movies 1, 2 also show this behavior of the single trials and other single trial characteristics. In addition, as expected, the volume of state space explored in response to the stimulus diminishes in projections of state space made by principal components as one goes from the first projections to PC7 and PC8 (Movies 3–5).

### Fixed-point attractor

**Figure 5:**
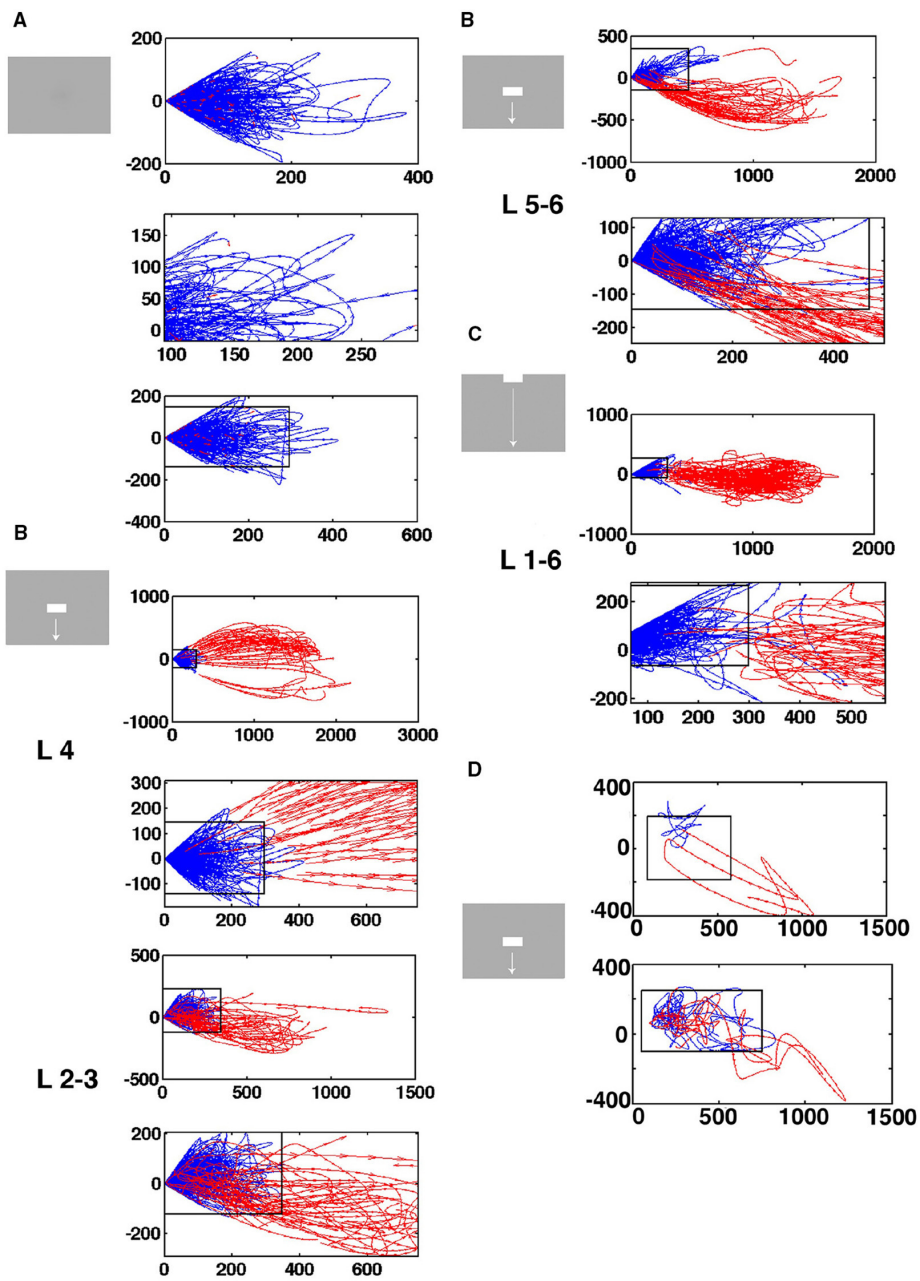
**Separatrices in all cortical layers**. Velocity vectors per ms in state space made by the first 2 principal components of the spiking from single electrode penetrations. **(A)** Spontaneous ongoing spiking. Note that all vectors at a certain distance bend from pointing outwards from the fixed point to point back to the fixed point from another angle. **(B)** Separatrices for bar moving down from CFOV. The separatrix also separates pre-stimulus velocity vectors pointing toward the fixed point from vectors showing flow in the opposite direction in evoked trials in the granular layer 30–50 ms after stimulus start (zoomed part of state space). Similar separatrices in layers 2–3 and 5–6 (**B** repeated). **(C)** Separatrices for bar moving down from peripheral FOV. Separatrix determined from collective spiking in all layers by diverging vector flows in pre-stimulus time and when trials became evoked 350–450 ms. **(D)** Top: Vector flow from 1 trial where the trajectory returns quickly toward the fixed point-for thereafter to be evoked again. Below: One evoked trial and two stuck stimulus trials. The values on the axes are those from the first (x-axis) and second (y-axis) principal components.

So far our results indicated that the spontaneous ongoing spiking when the visual FOV is devoid of stimuli and the spiking driven by the stimuli in the majority (*n* = 65) of electrode penetrations were qualitatively different and evolved in different parts of state space (Figures 1, 2; Movie 1). To examine the spontaneous spiking mathematically, we calculated the velocity vectors of the trajectories of each single trial by taking the temporal derivative at each single trial trajectory point (Materials and Methods). In this way we got the spiking vector flow fields separately for the granular supragranular and infragranular layers. As the area of the flow fields diminished in state space projections made of higher principal components (like in Movies 1–5), we show here only the flow fields in state space made of the first two components (Figure 5).

In the rest condition, the rest flow vectors, when they reach a certain distance from the fixed point, bend off and revert their direction to point toward the fixed point (0,0) (Figure 5A). Similar vector flows prevail in the pre-stimulus periods (blue vectors in Figures 5B–D). This shows that there is a fixed-point attractor at (0,0) attracting the spontaneous spiking and keeping it at a short distance. This result was confirmed in all 82 electrode penetrations (Data available at https://www.dropbox.com/sh/wxnksycscye1n6z/AAATK0Lq37KPgY4jEPmcF3p2a?dl=0).

### Separatrices in all cortical layers

In accordance with Huys et al. (2016), we suspected that the spiking state space is divided into two *mathematically separate domains*, within which two different dynamics take place: in one domain the spiking during the control condition (gray screen) and in the pre-stimulus interval (gray screen), i.e., the spontaneous ongoing spiking, evolves over time, in the other the visually evoked spiking evolves over time. If this is so, it must be documented that the evoked spiking dynamics in state space is mathematical separated from the spontaneous ongoing spiking by a border. This border is called a separatrix and is mathematically defined by diverging phase flows on either side. We calculated PCA state spaces separately for the leads covering the supragranular layers, the granular layer and the infragranular layers (Materials and Methods). In these state spaces we plotted the velocity vector flows.

At a distance from the fixed-point, most of the flow vectors of the stimulus trials pointed away from the fixed point 30–50 ms after the start of the stimulus (red vectors in Figures 5B–D). For stimulus conditions in which the stimuli and hence the local cortical dynamics only later drove spiking away from the fixed point the divergence of the flows happened later, for example at 350 ms as in the example in Figure 5C. However, a minority of stimulus trials the vectors bend off like the spontaneous ongoing spiking vectors and pointed toward the fixed point (Figures 5C,D). In average this happened in 40% of the trials from the electrodes mapping the CFOV (see later). In 17 of the electrode penetrations outside the zone mapping the CFOV, diverging vector flow was limited to 1 or 2 trials and in 2 penetrations the vectors bended off at short distances to the fixed point in all trials. In summary, 71 of the 82 electrode penetrations had a zone with diverging vector flows at a certain distance from (0,0) in each of the supragranular, granular and infragranular layers in one or more of the stimulus conditions.

At this border-zone, spontaneous ongoing spiking vectors and a proportion of vectors from stimulus trials bended off and pointed back toward the fixed point. In contrast, a smaller or larger large proportion of stimulus trial vectors, in a certain time interval, pointed away from the fixed point into the surrounding state space. This proved that there were diverging phase flows at a certain distance from the fixed point, and hence that a separatrix located at this distance separating spontaneous type dynamics from visual evoked dynamics.

As described in the methods, we estimated the localization of the separatrix. The rectangular boxes in Figures 5A–D show the estimated positions of the separatrix. Inside the separatrix the vectors pointed in all directions (Figure 5D). As one approaches the inside of the separatrix, more and more of the spontaneous spiking trajectories heading away from the fixed point bended off and their flow vectors then pointed again toward the fixed point (Figures 5A,B). Close to the inside of the separatrix the last of the single trial spontaneous spiking trajectories and their flow vectors bended off. For the trials crossing the separatrix, their vectors of their trajectories crossed the separatrix directed away from the fixed point (Figures 5B–D). In practice then, the estimated positions of the separatrices for each layer fitted reasonably well with the diverging vector flows. For example the separatrix also separated the pre-stimulus vector flows from the flow of the evoked trials (Figures 5B–D). The position, and hence the area of the rectangular box varied somewhat among the electrode penetrations (Supplementary Figure 3). However, the area was not correlated with the maximal number of trials escaping the space of the separatrix (*p* > 0.1, *t*-test on correlation coefficient, *r*^2^).

Instead of estimating the position of the separatrix, layer-by-layer in the cortex, we also calculated the velocity flow vectors for the collective spiking across all cortical layers. Two electrode penetrations did not have any trial with outward directed flows opposing the vectors pointing inwards, probably because the electrode position was too far from the populations mapping the moving objects. In these cases all trials were inseparable from the rest trials shown in Figure 5A, and no separatrix could be found. In 79 out of 82 electrode penetrations, we found a dynamical threshold, a separatrix for the collective spiking of all layers of the cortex. This separated chaotic-like trajectories from evoked trajectories (Figure 5C).

The rest spiking dynamics and the visual evoked dynamics therefore are two different dynamical states in the stimulus condition examples shown in Figure 5. As also apparent from Figure 5, there were no indications of other fixed points (velocity vectors converging toward a point in state space outside the separatrix), neither for the vector flow analysis separately of the granular, supragranular and infragranular layers (examples in Figure 5B), nor from the vector flows based on all layers (example in Figure 5C). All separatrices are available at https://www.dropbox.com/sh/wxnksycscye1n6z/AAATK0Lq37KPgY4jEPmcF3p2a?dl=0.

### Entry into the evoked states

The existence of the separatrix, and the qualitative differences in the behavior of the single trial dynamics, imply that one can divide trials into those showing spontaneous ongoing dynamics and those escaping the separatrix: In short, stock trials and evoked trials. The spiking in all layers can enter the visually evoked state either as driven by sharp transients or as smoothly increasing trajectory velocity (Figures 3–5).

**Figure 6:**
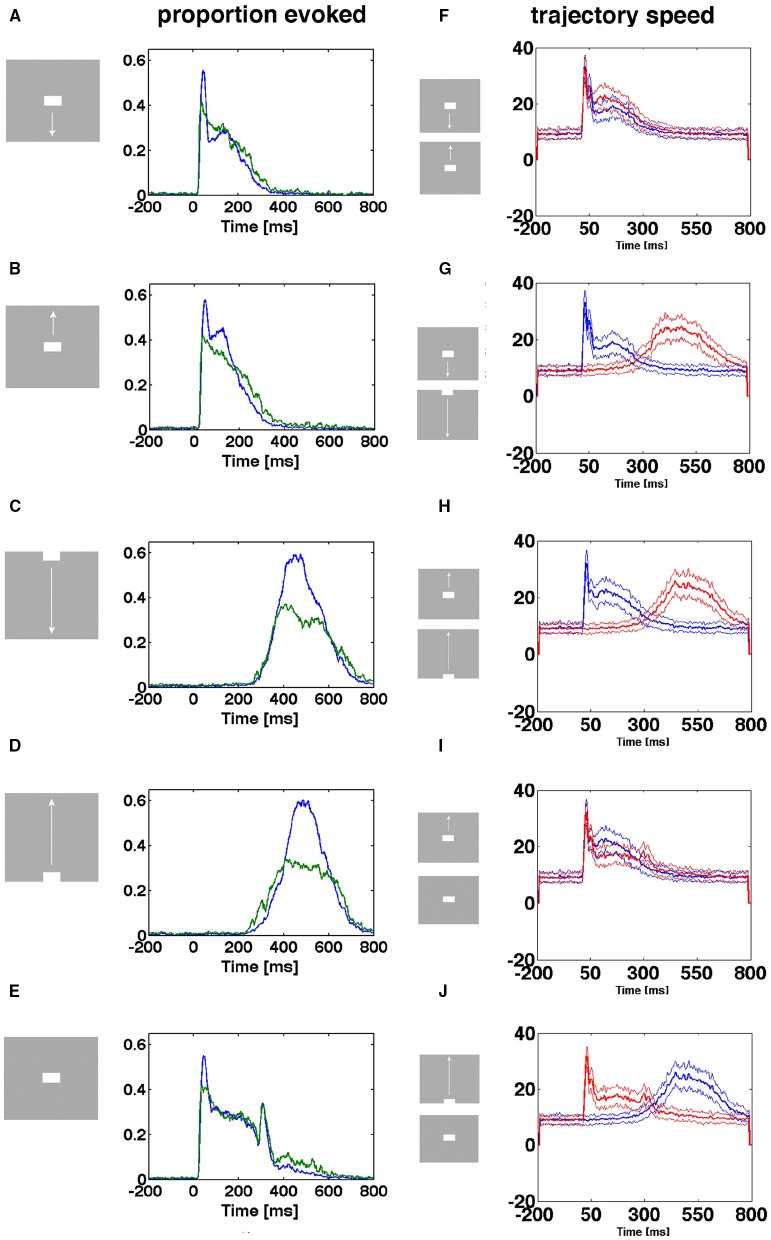
**Two ways of entering the evoked state**. Proportion evoked trials and trajectory speeds as functions of time from 17 electrode penetrations in the cortex mapping the CFOV. Stimulus start 0 ms. **(A,B,E)** Stimuli appearing in CFOV elicit sharp transients that swiftly brings the spiking into the evoked states. **(C,D)** For stimuli appearing outside the CFOV, the dynamics of the cortex initiate smoothly raising spiking dynamics gradually increasing trajectory speed and proportion evoked. Blue curves: mean proportion of trials evoked, green curves: square-root of variance. **(F)** Up and down moving bars appearing in CFOV give sharply increasing trajectory speeds. **(G,H,J)** Bars appearing first in peripheral field of view are mapped later with smoothly increasing trajectory speeds. **(I)** Stationary and moving bar appearing in CFOV have similar trajectory speeds. **(J)** A stationary bar and a bar passing the CFOV have different trajectory speeds. Note the correspondence between speeds and proportion-evoked trials. Mean thick curve, square root of variance thin curves.

When the proportion of evoked trials is plotted against time, indeed there were two types of entry. The stimulus conditions starting with a sharp visual transient change in the luminance and contrast, with a delay of 25–35 ms, increased the proportion of evoked trials suddenly from 0.005 to 0.6 (Figures 6A,B,E) for the population of neurons mapping the CFOV. In contrast, the same population of neurons took about 200 ms to achieve the same increase in proportion of evoked trials when they mapped an object moving toward and passing the CFOV (Figures 6C,D). The sharp and smooth modes of entering the evoked state were equally efficient with respect to the proportion of trials evoked (Figures 6A–E).

In the (beige colored in Figure 1) cortical transition zone mapping the edge of the CFOV there were trials of both types: some entered the evoked state rapidly, some more smoothly (Supplementary Figure 4). The electrode penetrations from the cortical zones mapping the peripheral FOV were only exceptionally mapping the exact part of the FOV in which the moving bar first appeared, so by far the most trials entered the evoked state in the smooth manner (Supplementary Figure 4). Generally for all cortical zones outside that mapping the CFOV, the proportion of evoked trials for the conditions in which the zone mapped the stimulus, was less than in the CFOV (Supplementary Figure 4).

The size of the flow vectors differed markedly between the rest condition and the stimulus conditions, showing that spontaneous ongoing spiking trajectories in general progress more slowly than the evoked trajectories (Figures 6F–J). The speeds as a function of time after the onsets of the transients did not differ between the sharp transient conditions, neither between the smooth entry conditions, but differed considerably between the sharp transient and smooth transient conditions (Figures 6G,H,J) (*p* < 0.01 with correction for family wise error). This difference in the dynamics of evoked trajectories is also obvious when Movies 1, 2 are compared. In the pre-stimulus period the trajectory speeds are moderate.

### Transition from slow chaotic-like trajectories to fast, smooth trajectories

**Figure 7:**
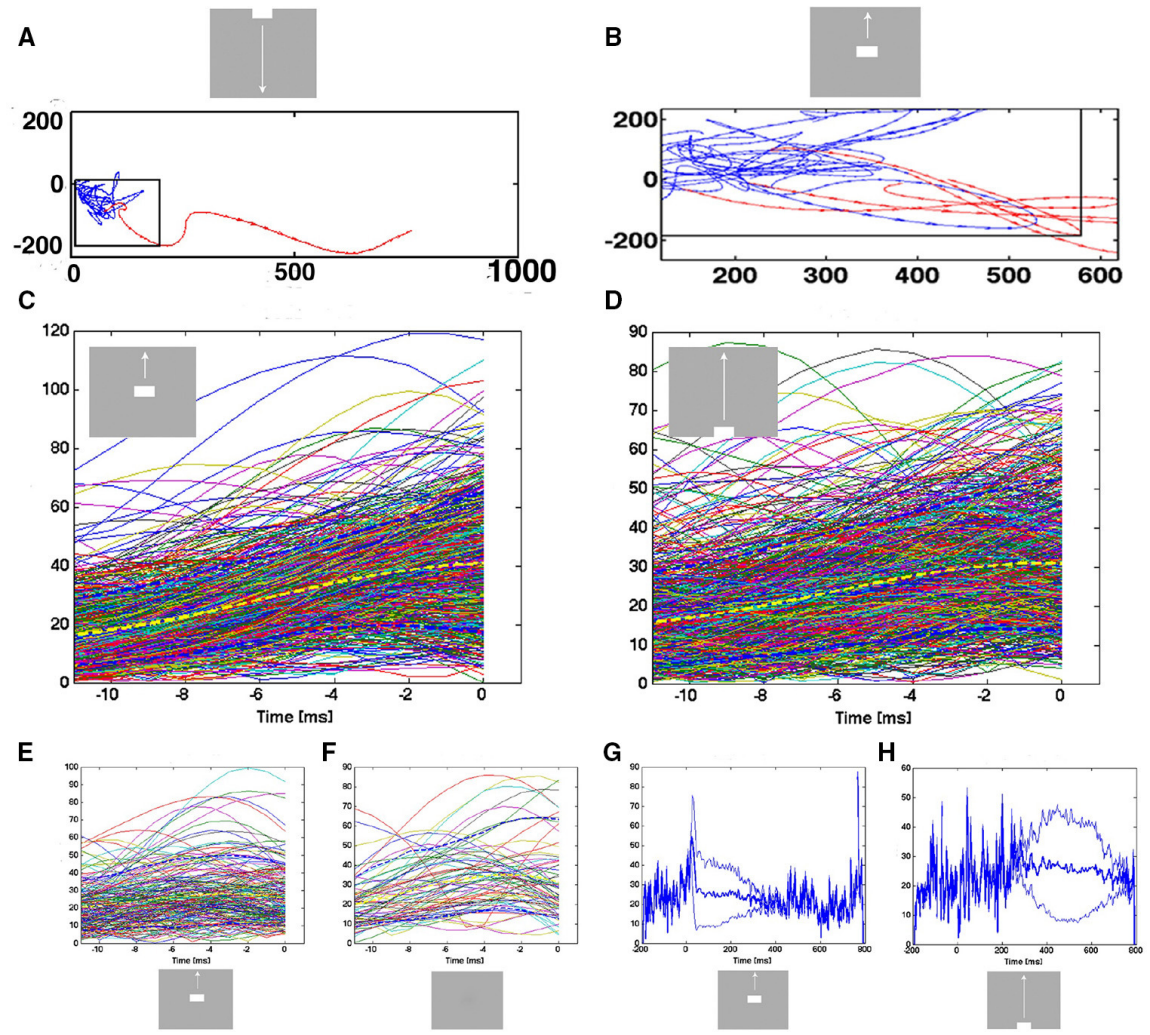
**Transitions from spontaneous spiking to evoked spiking: vector flow**. **(A)** Transition from chaotic-like vector flow to evoked vector flow at the blue-red transition (animal 2). **(B)** Two trial transitions at 25 ms and one re-entry. Note also the transition of one of the trials in the pre-stimulus period (blue vectors) bending off at the separatrix. **(C)** Acceleration of trajectory speeds prior to the first entry into the evoked state after 20 ms post stimulus. All evoked trials from the 17 electrode penetrations mapping the CFOV. Yellow: mean speed; dark-blue stippled lines: 10%-ile and 90%-ile. A few trials were evoked prior to the sharp transient. X-axis: time prior to the crossing of the separatrix. Y-axis speed in state space. **(D)** Smooth transient driven acceleration of trajectory speeds prior to the entry into the evoked state after 280 ms. **(E)** Re-entries into the evoked state after 280 ms. **(F)** Entries into the evoked state (after 220 ms) by trials in the control condition. **(G,H)** Mean trajectory speeds and square root of variance for all evoked trials in electrode penetrations mapping the CFOV. In the pre-stimulus interval, single trials enter the evoked state with high speeds.

In the conditions in which a sharp visual transient changes the visual scene, the flow vectors suddenly change from small and directed in shifting directions to large and smoothly taking a course out of the separatrix (Figure 5D, top, Figure 7A). This transition from erratic or chaotic-like trajectory to smooth directed trajectory always took place inside the separatrix, as evidenced in these conditions of the 80 electrode penetrations having a separatrix (*n* = 240). The position of where the transition takes place depends on the position of the trajectory when the transient hits (Figures 5, 7A,B). For the conditions in which the evoked state was initiated by a smooth transient, there was a similar transition from chaotic-like to smooth trajectories inside the separatrix (*n* = 160). The velocity vectors however enlarged more gradually (Figure 7B). Whereas the separatrix divided the state space into a spontaneous ongoing spiking domain and an evoked spiking domain on the basis of the direction of the vector flows, the transition from the spontaneous chaotic-like trajectory to the evoked faster and smooth trajectory was based on the length, smoothness and direction of the flow vectors.

Since the transitions started inside the separatrix, the acceleration of the vector flows should precede the crossing of the separatrix. Therefore, we aligned the time course of the trajectory speeds to the instant of the first passage of the separatrix after the stimulus onset, i.e., the first time the spiking became evoked. For the sharp transient conditions, the mean trajectory speed increased from about 15–40 in the ms prior to the instant the trial became evoked in the cortex mapping the CFOV (Figure 7C). For some trials, this acceleration started just 2 ms prior to the passage of the separatrix, for other trials the acceleration started 12 ms prior to the instant the trials became evoked.

For the trials entering the evoked state driven by smooth transients, the speed increased from 15 to 30 over a period of 4–12 prior to the passage of the separatrix, with individual variations (Figure 7D). Thus, irrespective of whether the transient that accelerated the speed was sharp or smooth, the trajectory speed increased just prior to its passage of the separatrix. This could be a causal relation, i.e., that the transition is a precondition for the evoked state. Further, a certain absolute speed of the trajectory might be needed to counteract the attraction from the fixed point; unless this speed is achieved, the spiking will not enter the evoked state. In fact, the few trials that became evoked prior to the effects of the sharp or smooth transients were almost all of speeds higher than 10 (state space units/ms) (Figures 7G,H).

### Single trial spiking can enter the evoked state several times

**Figure 8:**
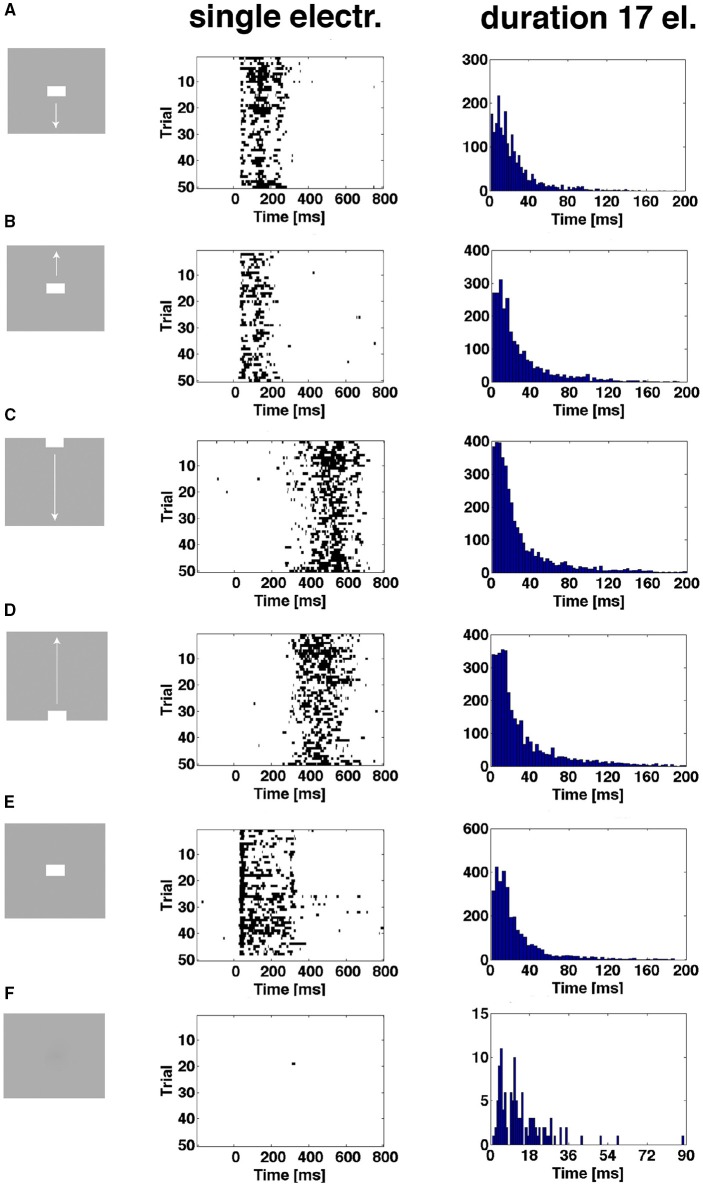
**The spiking enters the evoked state more than once**. First column shows the 6 experimental conditions. Second column shows single trial durations of epochs of evoked state for 3 electrode penetrations in the CFOV mapping zone, rows **(A,B,E)**, and 2 electrode penetrations from cortical zones **(A,B)** (Figure 3) for rows **(C,D)**. The evoked state is determined from the vector fields generated by the trajectories of all 16 leads (Materials and Methods). Epochs from 50 trials of one example electrode per panel row. Third column: distributions of durations of all evoked state epochs for all electrode penetrations (*n* = 17) in the CFOV mapping zone condition by condition. **(F)** Gray screen (rest), one trial enter the evoked state for a short period; all such entries were of short durations (last column). Supplementary Figure 6 show the original spike rasters from the example electrodes in the second column.

As shown in Figure 6 and Supplementary Figure 4, the proportion of trials being in the evoked state at a certain point in time was always less than 1.0, sometimes less than 0.2. Plotting the time intervals when the spiking of a trial was in the evoked state shows this for all conditions except the control. In addition, one trial can be in the evoked state several times. For the CFOV population of neurons, the short durations dominate the distribution of evoked time durations, irrespective of the stimulus condition (*p* > 0.1; *n* = 17) (Figure 8). This implies that a stationary object, when mapped, is not continuously represented by the same members of the population being in the evoked state for the duration of the stimulus (or until the OFF response). Table 2 shows the total number of trials becoming evoked, once or more times, in the post stimulus period. If one compares these numbers with the instantaneous proportion of trials evoked (Figure 6) it follows that not all trials were evoked at the peaks in Figure 6. This means that a trial not evoked at a peak can be evoked later (or earlier in the conditions in which the bars start in the peripheral FOV). Supplementary Figure 6 shows the original spike raster plots for all trials to compare with the conditions in Figure 8.

**Table 2:** **Number of trials evoked one or more times**.

**Condition**	**Lateral zone**	**Medial zone**	**Center**	**Edge**
Down from CFOV	145	674	715	303
Up from CFOV	620	522	730	398
Down	606	939	818	524
Up	576	901	819	552
Stationary bar	173	308	707	275
Gray screen	66	114	87	37
Total no. of trials	1000	1450	850	800

The durations of the single spiking trials varied from 1 ms to more than 150 ms.

This supports the theory that the fixed-point attractor continues to exist and exert its attraction even over evoked spiking states because the outgoing vector flows could be reversed at any time.

From Figures 6, 7 it is obvious that trials that become evoked several times are likely to have high trajectory speeds. The trajectory speed may be needed to overcome the attraction from the fixed point. As seen from Figures 5D, 7B, the trajectory speed may decrease and the trajectory returns to the state space inside the separatrix just to be accelerated again. For the trials becoming re-evoked, the trajectory speeds were generally above 10 and increased further somewhat prior to the spiking becoming evoked again (Figure 7E). However, the accelerations in these cases were moderate compared to those elicited by the fast transients (Figure 7). This behavior was consistent in all stimulus conditions (Supplementary Figure 5, Movies 1–5).

### The evoked states have similar behavior

As the trials could enter the evoked state either with a smoothly accelerating speed or with sharply accelerating speed one may think that they could continue to evolve differently in state space. In contrast, the durations they stayed evoked did not differ between conditions (Figure 8). However, the vector flows may differ in directions. There could be temporary unstable fixed points forming the vector flows. Furthermore, the vector flows may differ between conditions.

**Figure 9:**
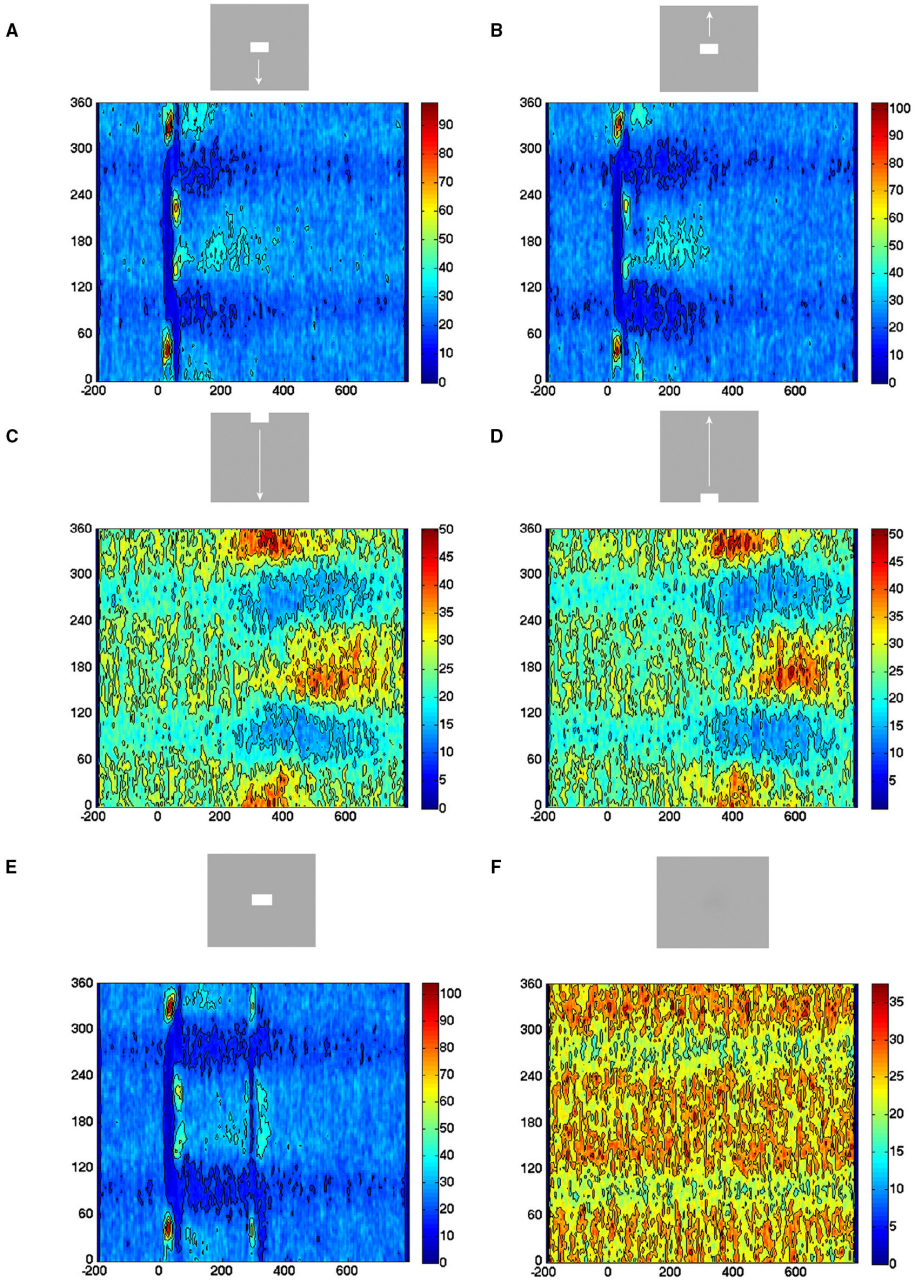
**All evoked states have similar exploration of state space**. Direction of the velocity flow vectors in state space. Y-axis: direction in degrees. X-axis: time in ms. Color scale: Number of trials with vectors pointing in a certain direction at a certain moment. **(A)** Vectors point in all directions in pre-stimulus interval. Post-stimulus, vectors point first away from fixed point (350–45°), then turning around (130–240°) and then, more slowly, back toward the fixed point (180°). **(B)** Similar chain of events. **(C)** More gradual change in the outwards and back to fixed point pointing directions. **(D)** Idem, **(E)** the on- and offset of the stationary square produce similar chain of events as in conditions **(A,B)**. **(F)** Vectors point in all directions.

For these reasons we calculated the direction of the velocity vectors (0–360°) in all single trials (*n* = 850) per condition for the electrode penetrations mapping the CFOV. In the rest condition, i.e., during spontaneous ongoing spiking, the vectors pointed in all directions. Similarly, in the time intervals prior to the evoked episodes, the patterns were the same (Figure 9). This is the picture expected when all vectors point toward a stable fixed point from all directions or outward from an unstable fixed point in all directions, or erratically change directions. However, immediately after the trials entered the evoked states, the direction was away from the fixed point (350–45°) for shortly thereafter to reverse (160–200°) for the stimulus conditions starting with a sharp transient (Figures 9A,B,E). The return state lasted somewhat longer, in accordance with the somewhat slower return to the separatrix area. For the stimulus conditions smoothly entering the evoked states, the directions were identical, i.e., outwards 350–45° and returning 160–200° albeit not so sharply separated in time (Figures 9C,D).

These results speak against any additional fixed points, stable or unstable, directing the flows in the evoked part of state space. In addition, the stimulus conditions did not differ systematically with respect to direction (Figure 9). The results from the other cortical zones confirmed this (data not shown).

### Progression of the evoked state through cortical layers

In the primary visual cortex of carnivores and primates, the retinal spike trains via the lateral geniculate nucleus first change spiking in the granular layer. The spiking in the granular layer in turn changes the spiking in the supragranular layers, and in turn the neurons of the supragranular layers alter the spiking of the neurons in the infragranular layers. Infragranular layer neurons in turn are supposed to affect the spiking in the granular layer (Douglas et al., 1989; Binzegger et al., 2004).

**Figure 10:**
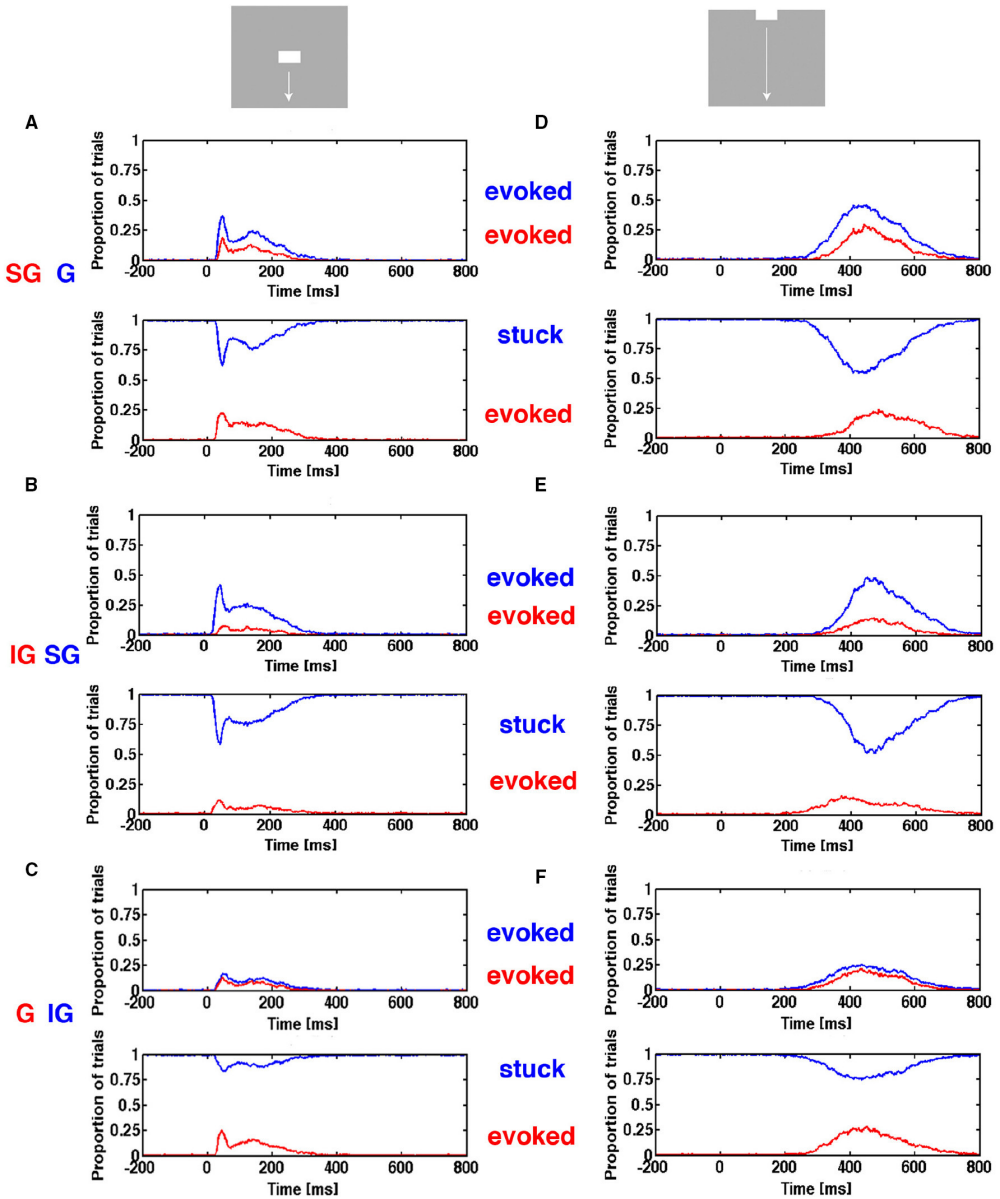
**Progression of evoked state through cortical layers**. The proportion of trials in the evoked state and proportion of stimulus trials stuck as function of time. **(A)** Top, the red curve is the trials evoked in supragranular layers (SG) *given* that the same trials were evoked in the granular layer (G) (blue curve). Below the trials evoked in the supragranular layers (red curve), *given* that the trials were stuck in the granular layer (blue curve). **(B)** (top) Trials evoked in the infragranular layers (red) (IG) given same trials evoked in supragranular layers (blue). Below: trials evoked in the infragranular layers, given trials stuck in supragranular layers (blue). **(C)** (top) Trials evoked in the granular layers (red) given same trials evoked in infragranular layers (blue). Below: trials evoked in the granular layers, given trials stuck in infragranular layers (blue). **(D)** as **(A,E)** as **(B,F)** as **(C)**.

In order to explore if this “canonical” sequence of spike changing dynamics was present in our data, we calculated the trajectory progressions in state space separately for the supragranular electrode leads, the granular leads and the infragranular leads (Materials and Methods) and asked the following questions: Neglecting a possible delay in the evolution of evoked state between layers, given that the spiking in a single trial was evoked in the granular layer at a given time after the stimulus, was the spiking in that trial also simultaneously evoked in the supragranular layers? Similarly, given that the spiking of the trial was evoked in the supragranular layers was the spiking also evoked in the infragranular layers? Calculated over all single trials for a condition, one gets estimates of the conditional probability of the dependence of the evoked state from layer to layer ms by ms (Figure 10).

As seen for the two conditions shown in Figure 10, the estimates of conditional probabilities are low (red curves in top panels). Note that the probability of being evoked in a layer is generally smaller than the probability of the collective spiking dynamics of the neurons in all layers being evoked (compare Figure 6 and Figure 10). The number of trials stuck in the non-evoked, spontaneous state is considerable. So we estimated in addition the conditional probability that given a trial stuck in the granular layer, what was the probability that it was evoked in the supragranular layer? Similarly, we estimated the probability that a trial stuck in supragranular layer was evoked in infragranular layer, and that a trial stuck in the infragranular layer was evoked in the granular layer as measures of independence of the dynamics between the layers. The conditional evoked probabilities were sometimes smaller than the conditional probabilities of trials stuck in one layer became evoked in the layer receiving the input from the stuck neurons (Figure 10). We analyzed other conditions and combinations of layers as well (data not shown) with the same general result. One should be careful by the interpretations of these conditional probabilities. For example that the trials evoked in the infragranular layers were with few exceptions also evoked in the granular layer could be because trials in the granular layer had a much higher probability of being evoked that had the same trials in the infragranular layers.

### Neither single trial spike rates nor single trial temporal dynamics distinguish stimulus conditions

So far we described the temporal dynamics of the spiking associated with different simple visual scenes. Perception of a visual scene is not a matter of statistics derived from a large number of trials with exposure of the same scene. Rather the perception of the scene is the result of a single and short exposure to one particular scene. This raises the question of whether the temporal course of spike rates from a single trial in a population of neurons mapping a particular part of the FOV is able to select which scene is exposed. An alternative formulation is whether the evoked trajectories of the same population are able to select which scene is exposed.

**Figure 11:**
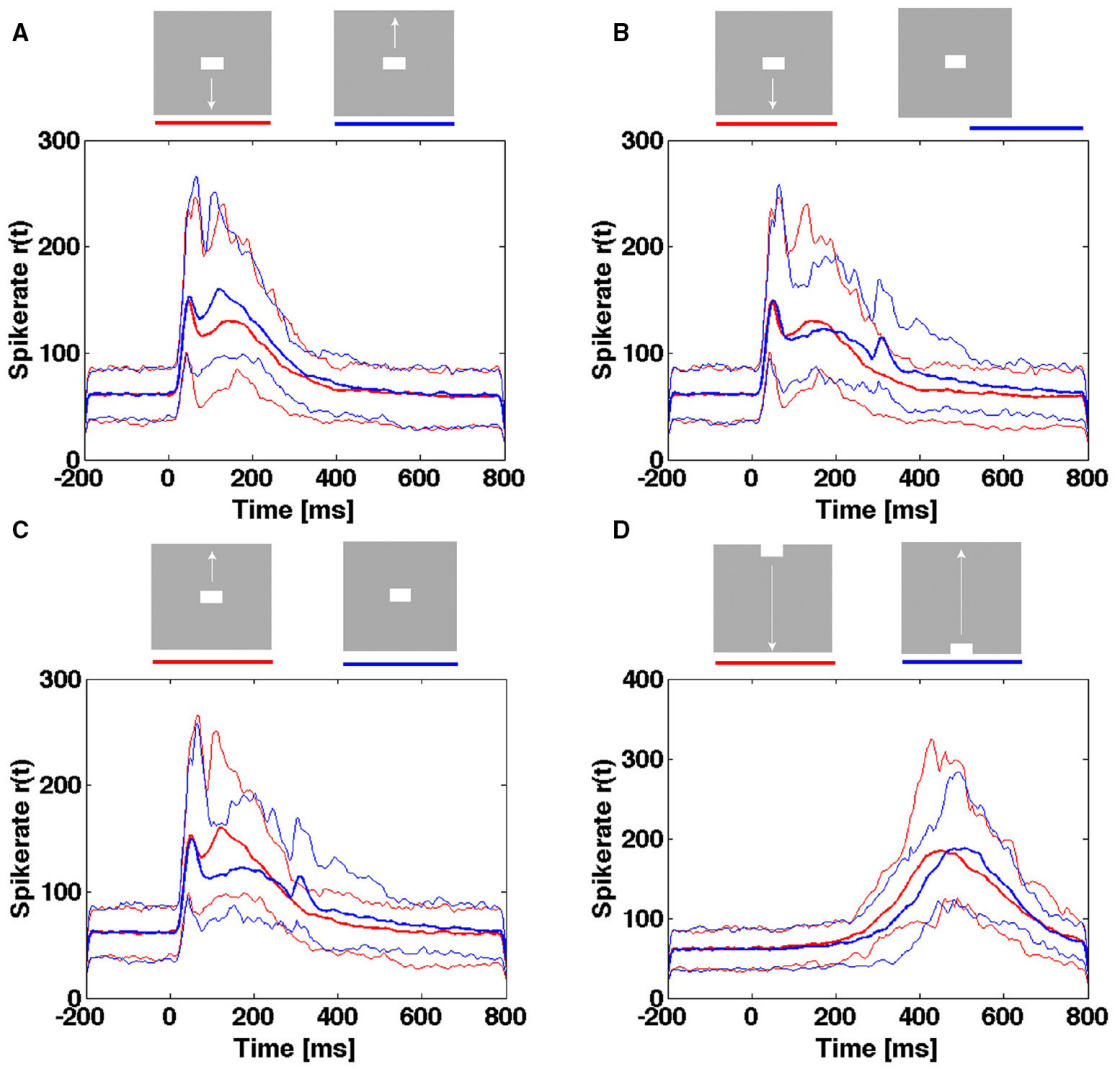
**Spiking rates do not distinguish stimulus conditions in single trials**. Multi-unit rates as function of times for 850 trials of the population mapping the CFOV. Mean: thick curves, 10%- ile and 90-%ile: thin curves. **(A)** Spike rates in Hz from trials with bar moving down (red curves) from CFOV vs. spike rates from trials with bar moving up (blue curves) from CFOV. **(B)** Spike rates from trials with bar moving down (red curves) from CFOV vs. spike rates from trials with stationary bar (blue curves) flashed in CFOV. **(C)** Spike rates from trials with bar moving up (red curves) from CFOV vs. spike rates from trials with stationary bar (blue curves). **(D)** Spike rates from trials with bar moving down (red curves) vs. bar moving up from peripheral FOV (blue curves).

For the population of neurons mapping the CFOV, they may from the rates of a single trial be able to distinguish objects moving across the CFOV from objects appearing in the CFOV (Figure 11). However, the temporal course of the spike rates of a single trial rate will not tell an object appearing in the CFOV and moving upwards from an object appearing in the CFOV and moving downwards or even a stationary object appearing in the CFOV (Figure 11). Neither is the temporal course of the spike rates able to distinguish an object passing the CFOV moving downwards from an object passing the CFOV moving upwards (Figure 11).

Next, we tested whether the single trial evoked trajectories were able to distinguish the 5 scenes with stimuli. The result was comparable with the spike rate analysis. The single trial trajectories only differed significantly at the time of the OFF response and thus (in a probabilistic sense) distinguished a still moving bar from the disappearance of a stationary bar at the CFOV (Table 3).

**Table 3:** **Epochs of difference in stimulus evoked spiking trajectories at cortex mapping CFOV**.

**Conditions compared**	**Supragranular**	**Granular**	**Infragranular**
Down from CFOV vs. Stat. bar	n.s	200–600	n.s.
Up from CFOV vs. Stat. bar	300–700	300–350	n.s
Up vs. down from CFOV	n.s.	n.s.	n.s
Bar moving up vs. moving down	n.s.	n.s.	n.s.

*Epochs significant at p < 0.05; n.s. not significant. All values in ms from start of stimulus*.

## Discussion

We provided evidence that the temporal dynamics of the spiking, in single trials, in the networks of the granular, supragranular and infragranular layers of the visual cortex during constant exposure to a gray screen evolved with rather slow chaotic-like trajectories around a fixed point. These trajectories are qualitatively indistinguishable from the trajectories of spontaneously evolving spiking in total darkness described by Huys et al. (2016). The granular, supragranular and infragranular layers each have a dynamical threshold, a separatrix, mathematically separating state space into the domain for the spontaneous spiking dynamics and the domain containing of evoked spiking. Sharp and smooth visual transients can drive the conversion of spontaneous chaotic-like spiking trajectories into higher speed smooth evoked trajectories. This conversion took place within the spontaneous spiking domain. The evoked spiking dynamics was either fast accelerating vector flow driven by fast transients or a slowly accelerating vector flow driven, in part, by smooth transients. Eventually, the flow in the evoked state space domain decreased and the trajectories always returned by another route to the state space occupied by the spontaneous spiking dynamics. The temporal dynamics was similar in supra-, granular and infra-granular layers and similar to the dynamics of the collective spiking in all cortical layers. However, whereas the temporal dynamics in single trials separated visual scenes with smooth transients from scenes with fast transients, it did not distinguish scenes composed by fast transients or scenes composed by smooth transients.

So if neither the dynamics, nor the time course of the original spike rates were able, in single trials, to distinguish a visual scene with an object moving from the CFOV from a scene with a stationary object flashed in the same position (Figure 11, Table 3), what then is the advantage of the dynamical analysis? The dynamical description is a complementary description of the spiking of single neurons and groups of neurons. The dynamics describe mathematically, succinctly the behavior of single neurons (Mazor and Laurent, 2005; Huys et al., 2016), the collective behavior of multi-units, small groups of neurons, neurons in one layer (Huys et al., 2016), and, in our case, the behavior of neurons across cortical layers, and of larger groups of simultaneously recorded neurons with multi-electrode arrays (Shenoy et al., 2013). It is difficult to achieve such mathematical descriptions of the cooperative spiking of many neurons from analyses of spike rates or spike timing. The results that the collective spiking dynamics across layers reproduced the temporal dynamics in granular, supragranular and infragranular layers show that the temporal dynamics described in the first paragraph apply to a network of neurons. This also means that the separatrix and fixed point are dynamical properties of the network of simultaneously recorded neurons.

We found no other fixed points in state space, and the vector flows of all evoked trials after a while, at shorter or longer distances from the separatrix, bended and pointed toward the fixed point again (Figures 5, 7, 9) Furthermore, the time spent evoked ranged from 1 ms to over a 100 ms (Figure 8) indicating that the attraction from the fixed point was stable over time even in the evoked state. This supports the conclusion that the network of neurons in visual areas 17 and 18 behaves as a mono-stable system with one stable fixed-point attractor and a separatrix. This mathematics predicts that all spiking trials, when evoked, will return to the neighborhood of the fixed point by another route than that by which the escaped the attraction as seen also in Movies 1–5.

The spontaneous ongoing spiking was a product of the autonomous dynamics of cortical network in areas 17 and 18 when the anesthetized animals were exposed to a homogenous gray screen of low luminance or exposed to total darkness. The autonomous dynamics of the brain may very likely produce other types of ongoing spiking in awake animals. We described the spontaneous ongoing spiking, in supragranular, granular and infragranular layers evolving as slowly progressing with vectors shifting direction frequently, as “chaotic-like.” We did not have sufficient amount of spiking data to substantiate that the trajectories indeed were chaotic, hence the term “chaotic-like.” Networks with recurrent excitatory connections and tightly balanced excitation and inhibition produce chaotic spiking (van Vreeswijk and Sompolinsky, 1996). The chaotic fluctuations of the membrane potential secures that the balanced network will react fast to external input.

The separatrix is a second (dynamical) threshold of networks of neurons *in vivo*. This distinguishes the area 17, 18 network from a classical balanced network as described by van Vreeswijk and Sompolinsky (1996) To escape the fixed-point attraction, the system needs either an external or internal (autonomous) driving force. In our preparation, the only external candidates are the visual transients and the spiking they cause. The sharp transient increased the spiking in the granular layer 20–30 ms after the visual transient. In single trials this coincides with the transition of the trajectory from chaotic-like to smooth and directed outwards trajectory in the granular layer (Figure 5B). From the transition, the trajectory speed accelerated within a few ms to near maximal speed with which the trajectory crossed the separatrix (Figures 3–5, Movies 1–5). The areas 17, 18 thus reacted fast to external input, despite the separatrix. Most likely, the sharp visual transient drove the transition.

The separatrix divided state space mathematically in two parts based on the vector flows. Therefore, dynamically, we refer to the spiking as in evoked state when the trajectory and vector flows are in the evoked part of state space. Although the separatrix is a network property it will also affect single neurons *in vivo*. The effect of the second threshold is to hamper the progression of the spontaneous spiking (Figures 5, 8 see also Huys et al., 2016). The second threshold may also hamper weak visual transients from driving the spiking to cross the threshold (Figures 5, 6, 7, Movies 1–5). The second threshold separates state space into evoked spiking and spontaneous spiking domains, which makes it easy to discriminate the two.

As the lateral geniculate after 120 ms no longer transmit ON transients but more smoothly increasing spiking transients to objects moving in the FOV (Lesica and Stanley, 2004; Jones et al., 2015), the neurons in the granular layer react accordingly (Harvey et al., 2009). One may wonder then how it is possible for the neurons to escape from the stable fixed point after 120 ms. At a certain speed, however the spontaneous spiking trajectory of the smoothly increasing spiking transients shifted to a slowly accelerating trajectory, that could cross the separatrix outwards and inwards several times, but eventually became outwards directed and explored state space to same extent as the other stimulus conditions (Figures 3, 4, 5C, 7). This behavior, most likely, was a consequence of diverse forms of automatic dynamics in combination with an external drive by the retinotopical input from the lateral geniculate nucleus. Harvey et al. (2009) described a predictive depolarization stretching 8° ahead of the cortical mapping of the moving object. Simultaneously with this, neurons, especially in the infragranular layers, started to fire in area 17/18 far ahead of the retinotopical mapping and hence ahead of the granular layer input (Figure 3 in Harvey et al., 2009). This took place some 140–220 ms after the start of motion. At 200 ms the neurons started to become evoked in the infragranular layers (Figure 10). Thus, several neurons along the path mapping the moving bar may already be in evoked states prior to the time the bar is actually mapped (Figures 6C,D; Harvey and Roland, 2013). The spiking then smoothly escapes the fixed-point 230–250 ms in all three layers (Figures 6–8) with moderate velocities. The velocities then may accelerate when the retinotopical input from the lateral geniculate drive the population of neurons. Similarly, when the neurons re-entered the evoked state after their initial escape, they seemed to keep their trajectory velocities above 10, which accelerated just prior to the re-entry (Figure 7).

We did not have any histological verification of the single electrode tracks though the cortical layers, but only the current source densities to give us an estimate of the position of layer 4, the granular layer (Supplementary Figure 1). With these cautions, one may conclude that the evoked states in a proportion of trials propagate from granular to supragranular layers, and that a small proportion of evoked states propagate from supragranular to infragranular layers. Further that the small proportion of trials evoked in infragranuar layers were also evoked in granular layers, but the reverse was not true (data not shown). However, a significant proportion of trials stuck in one layer were evoked in the presumed recipient layer, indicating that the transition from the spontaneous trajectory to evoked trajectory must have been driven by other neuron sources, local or remote. Notably, the deviance from canonical to “independent” evoked states was present from the time of the initial transient (Figure 9).

The temporal dynamics in single trials distinguished fast visual transients from slow visual transients (as did also the original spike rates). Our conclusion that neither single trial spiking rates from one electrode penetration nor a population of electrodes mapping the CFOV were able to distinguish the visual scenes was based on the laminar collective multiunit spiking in each of the granular, supragranular and infragranular layers. This disqualifies the multi-unit rate as a useful variable, but says nothing about possible subpopulations having directional sensitivities and nothing about whether the spiking contains (hitherto undiscovered) codes identifying the scenes. Similarly, the temporal dynamics of the single trial trajectories and the parts of state space they explored were insufficient to distinguish the visual scenes. The temporal evolution in state space of the vectors and single trial trajectories had the advantages that it could identify an attractor and a network threshold and describe spiking at different scales from the single neurons to the population across layers as a mono-stable excitable system. This implies that we can predict the temporal dynamics of the neurons. For example, that the population of neurons in the primary visual areas (17 and 18) needs not to re-organize its dynamics by creating new attractors when perturbed by visual transients. For example, that for any visual transient, the network of spiking neurons will always return to the spontaneous ongoing spiking state by another route than that it escaped. This shows that the spiking network in areas 17 and 18 behaved as a complex dynamical system. The information in pure temporal spike dynamics may be limited (Ganguli et al., 2008). Separating temporal from spatial dynamics is artificial and illogical. The space-time dynamics describing how the spatial interactions of spiking neurons evolve in brain networks is likely to efficiently separate visual scenes.

## Author contributions

PR and MH designed the experiments; LF, LB, and PR designed and did the analysis of data. PR and LB wrote the final versions of the manuscript.

## Funding

This work was supported by the Danish Ministry for Science and Innovations and the Dynamical Systems Interdisciplinary Network.

### Conflict of interest statement

The authors declare that the research was conducted in the absence of any commercial or financial relationships that could be construed as a potential conflict of interest.

## Abbreviations

FOV: field of view
CFOV: center of field of view.

## References

[B1] Benjamini Y. Hochberg Y. (1995). Controlling the false discovery rate- a practical and powerful approach to multiple testing. J. R. Stat. Soc. B Methodol. 57, 289–300.

[B2] Binzegger T. Douglas R. J. Martin K. A. C. (2004). A quantitative map of the circuit of cat primary visual cortex. J. Neurosci. 24, 8441–8453. 10.1523/JNEUROSCI.1400-04.2004 15456817 PMC6729898

[B3] Brette R. (2015). Philosophy of the spike: rate-based vs. spike-based theories of the brain. Front. Syst. Neurosci. 9:151. 10.3389/fnsys.2015.00151 26617496 PMC4639701

[B4] Douglas R. Martin K. A. C. Whitteridge D. (1989). A canonical microcircuit for neocortex. Neural Comput. 1, 480–488. 10.1162/neco.1989.1.4.480

[B5] Ganguli S. Huh D. Sompolinsky H. (2008). Memory traces in dynamical systems. Proc. Natl. Acad. Sci. U.S.A. 105, 18970–18975. 10.1073/pnas.0804451105 19020074 PMC2596211

[B6] Harvey M. A. Roland P. E. (2013). Laminar firing and membrane dynamics in four visual areas exposed to two objects moving to occlusion. Front. Syst. Neurosci. 7:23. 10.3389/fnsys.2013.00023 23805082 PMC3691547

[B7] Harvey M. A. Valentiniene S. Roland P. E. (2009). Cortical membrane potential dynamics and laminar firing during object motion. Front. Syst. Neurosci. 3:7. 10.3389/neuro.06.007.2009 19753323 PMC2742661

[B8] Hubel D. H. Wiesel T. N. (1959). Receptive fields of single neurons in the cat‘s striate cortex. J. Physiol. 148, 574–591. 10.1113/jphysiol.1959.sp006308 14403679 PMC1363130

[B9] Huys R. Jirsa V. K. Darokhan Z. Valentiniene S. Roland P. E. (2016). Visual evoked spiking evolves while spontaneous ongoing dynamics persist. Front. Syst. Neurosci. 9:183. 10.3389/fnsys.2015.00183 26778982 PMC4705305

[B10] Jones H. E. Andolina I. M. Shipp S. D. Adams D. L. Cuderio J. Salt T. E.. (2015). Figure-ground modulation in awake primate thalamus. *Proc. Natl. Acad. Sci*. U.S.A. 112, 7085–7090. 10.1073/pnas.1405162112 25901330 PMC4460497

[B11] Jung R. (1959). Microphysiologie corticaler Neurone: Ein Beitrag zur Koordination der Hirnrinde und des visuellen Systems, in Structure and Function of the Cerebral Cortex, eds Tower D. B. Schadé J. P. (Amsterdam: Elsevier), 204–233.

[B12] Lesica N. A. Stanley G. B. (2004). Encoding of natural scene movies by tonic and burst spikes in the lateral geniculate nucleus. J. Neurosci. 24, 10731–10740. 10.1523/JNEUROSCI.3059-04.2004 15564591 PMC6730113

[B13] Mazor O. Laurent G. (2005). Transient dynamics versus fixed points in odor representations by locust antennal lobe projection neurons. Neuron 48, 661–673. 10.1016/j.neuron.2005.09.032 16301181

[B14] Rappelsberger P. Pockberger H. Petsche H. (1981). Current source density analysis: methods and application to simultaneously recorded field potential of the rabbit's visual cortex. Phlügers Arch. 389, 159–170. 10.1007/BF00582108 6259585

[B15] Reid R. C. Victor J. D. Shapley R. M. (1997). The use of m-sequences in the analysis of visual neurons: linear receptive field properties. Vis. Neurosci. 14, 1015–1027. 10.1017/S0952523800011743 9447685

[B16] Shenoy K. V. Sahani M. Churchland M. M. (2013). Cortical control og arm movements: a dynamical systems perscepctive. Annu. Rev. Neurosci. 36, 337–359. 10.1146/annurev-neuro-062111-150509 23725001

[B17] van Vreeswijk C. Sompolinsky H. (1996). Chaos in neural networks with balanced excitatory and inhibitory activity. Science 274, 1724–1726. 10.1126/science.274.5293.1724 8939866

