# Supplementary material for: The Second Spiking Threshold: Dynamics of Laminar Network Spiking in the Visual Cortex
Source: Front Syst Neurosci. 2016 Aug 17;10:65. doi: 10.3389/fnsys.2016.00065 (PMC4987378; doi:10.3389/fnsys.2016.00065)
Supplement: Supplementary file 6 [file DataSheet1.docx]

**Supplementary materials**

**
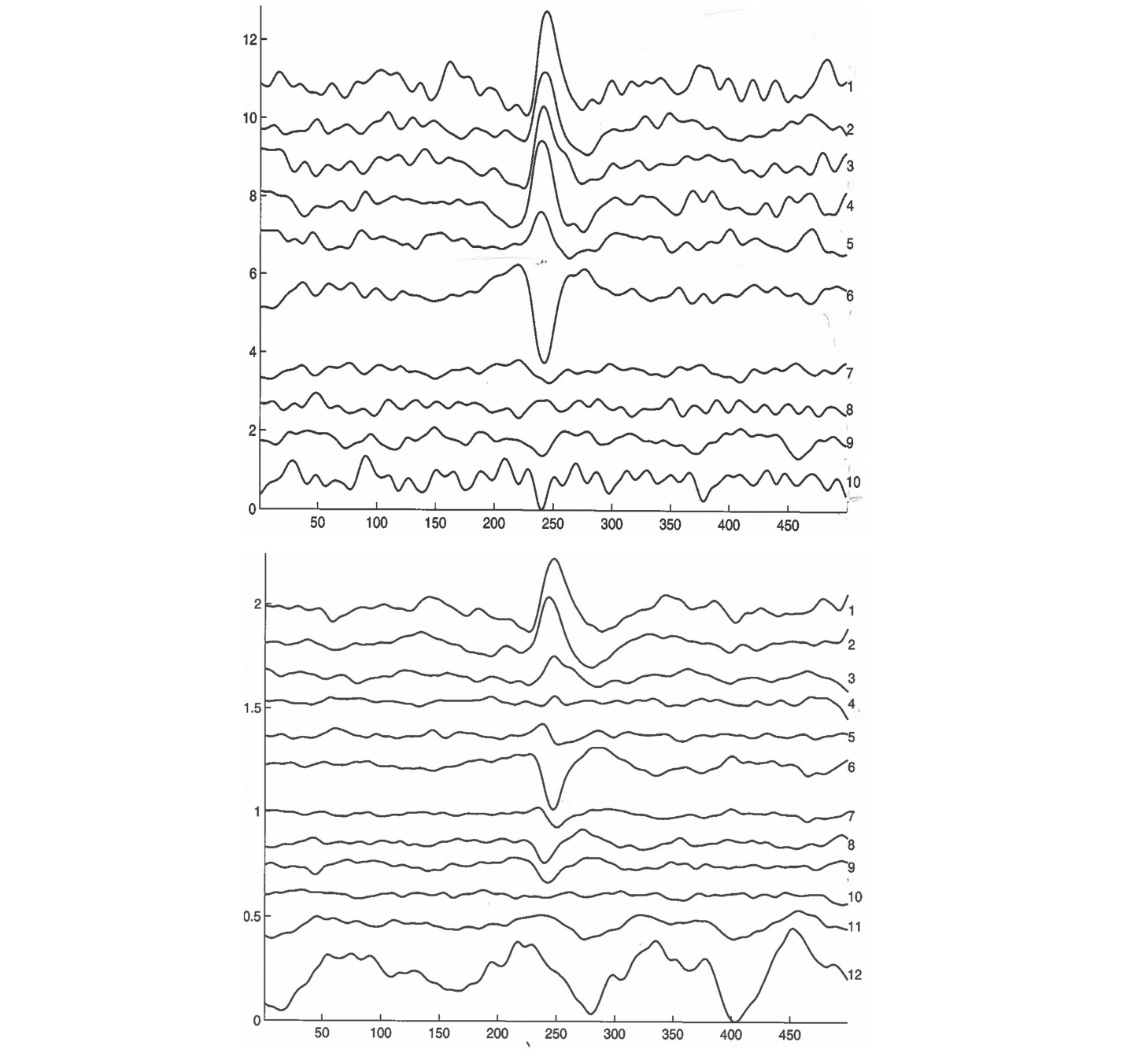
**

**SFig. 1** *Current source densities*

Only the top 10 respective 12 leads are shown. (Animals 5 and 6). Leads numbered from the cortical surface.


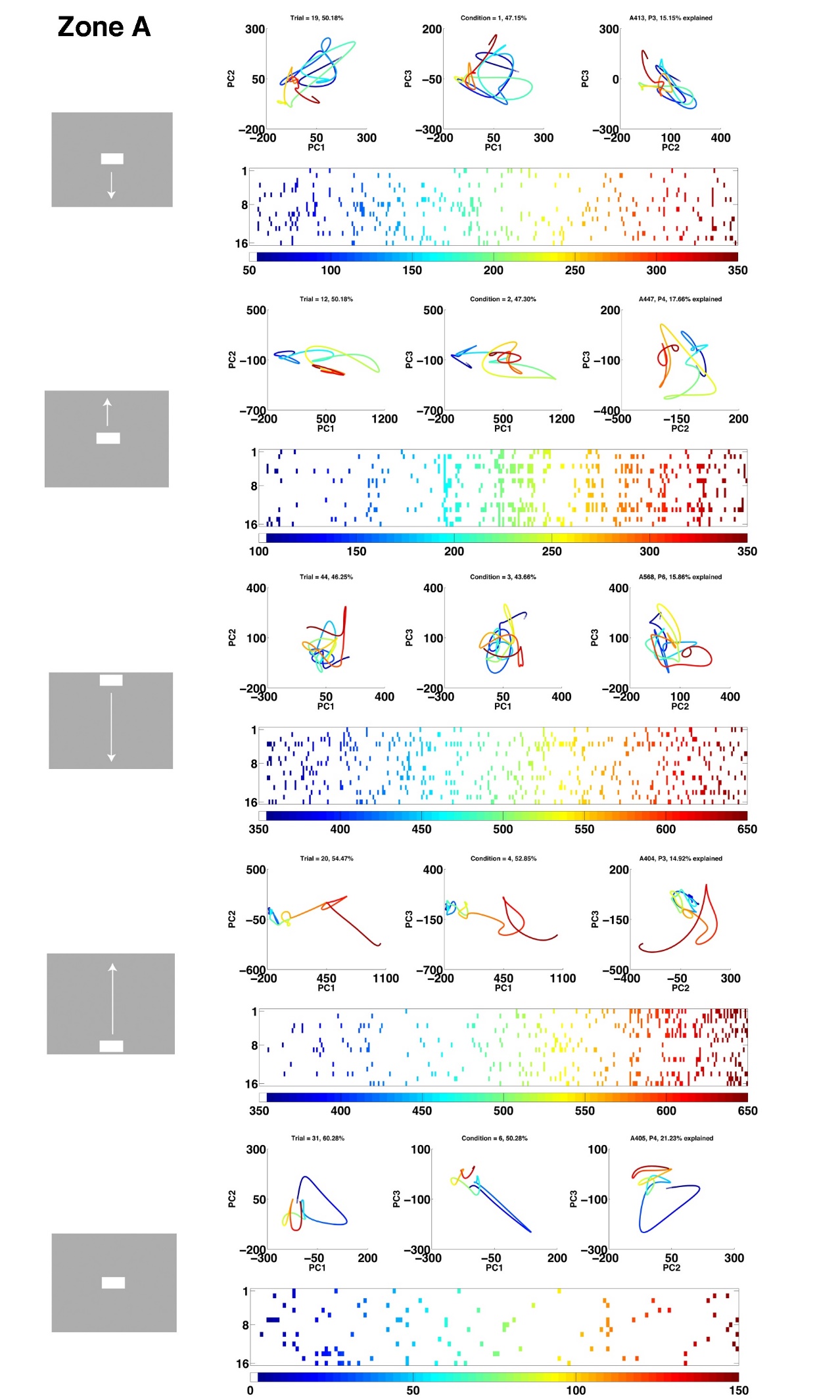


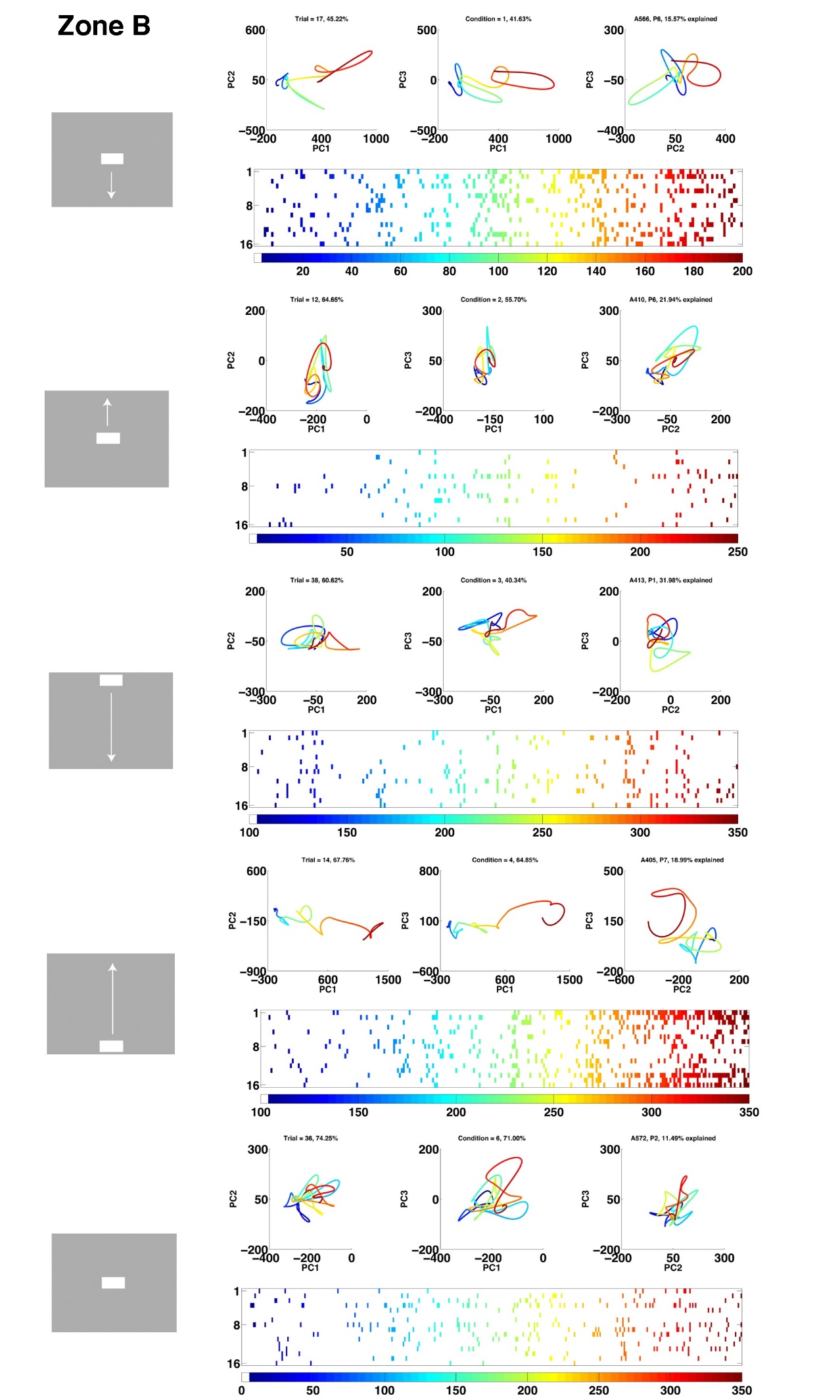


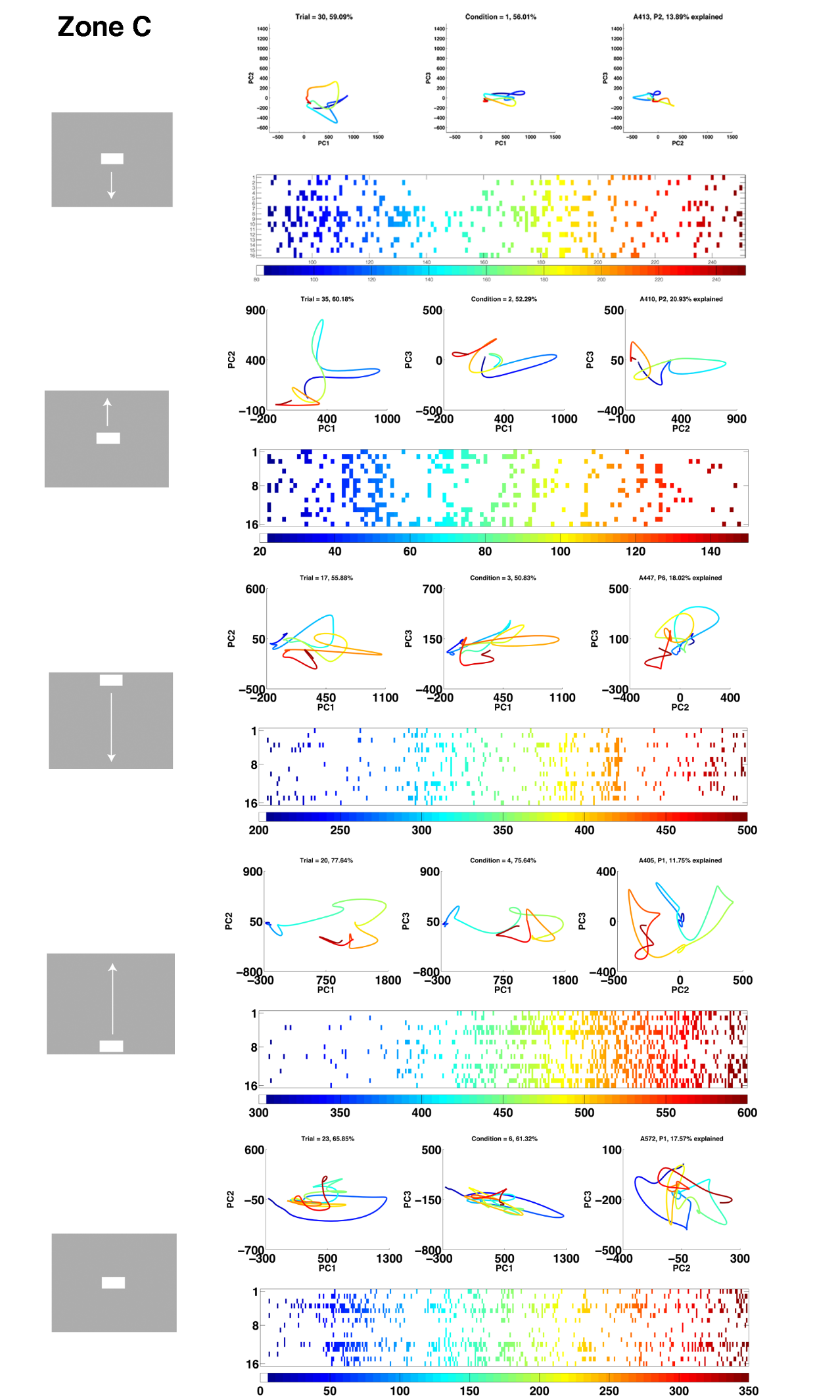


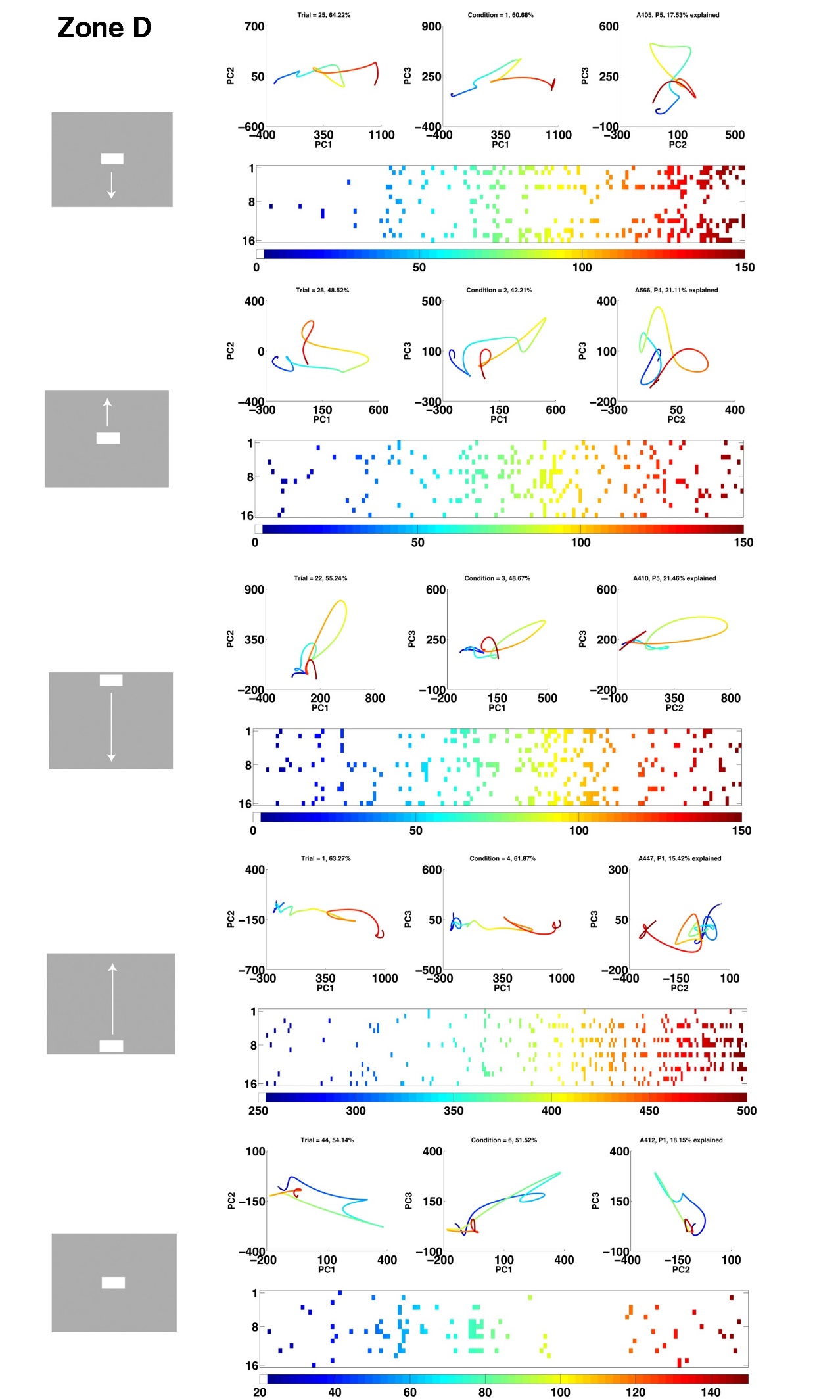


**SFig2** *Additional single trial trajectories in state space with raw spike rasters from the same trial.* The cortical zones are those in Fig.3. Three projections of the 3-dimensional state space as in Fig. 4. Other conventions as in legend to Fig. 4. i.e. the color coding of time is identical in trajectories and the single spike rasters. *.*

*
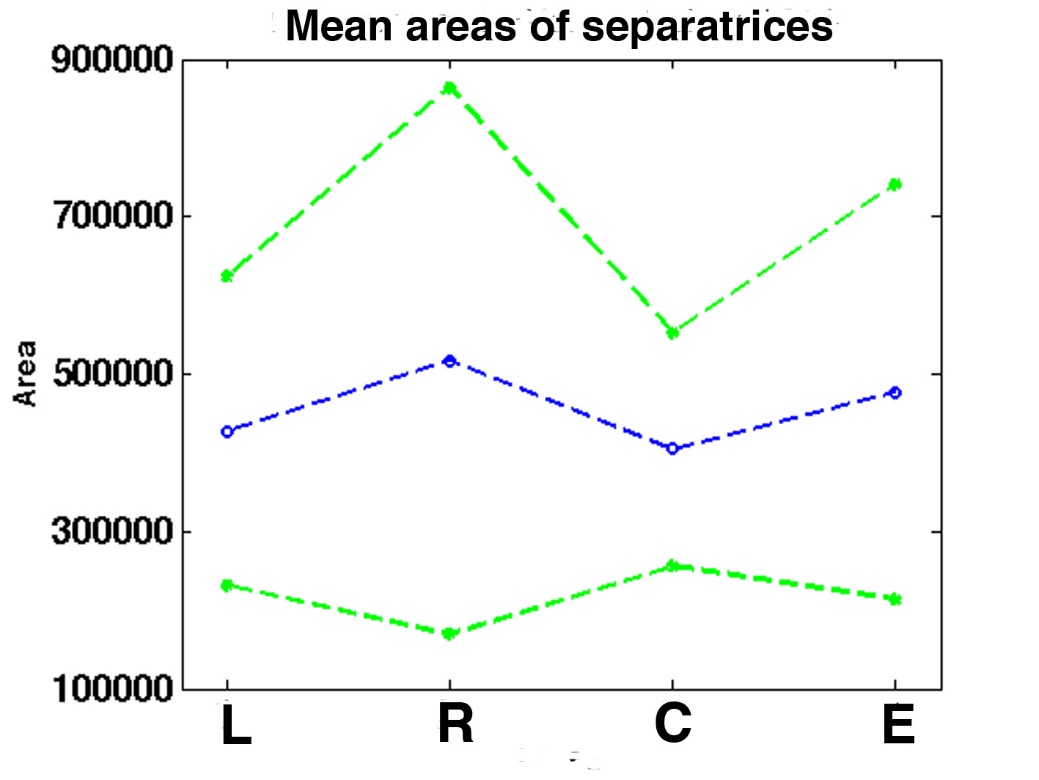
*

**SFig. 3** *Areas of the separatrices*

From the 4 cortical zones. L:_left, R : right; C. center, E: edge equal to zones A,B,C,D of Fig. 3.


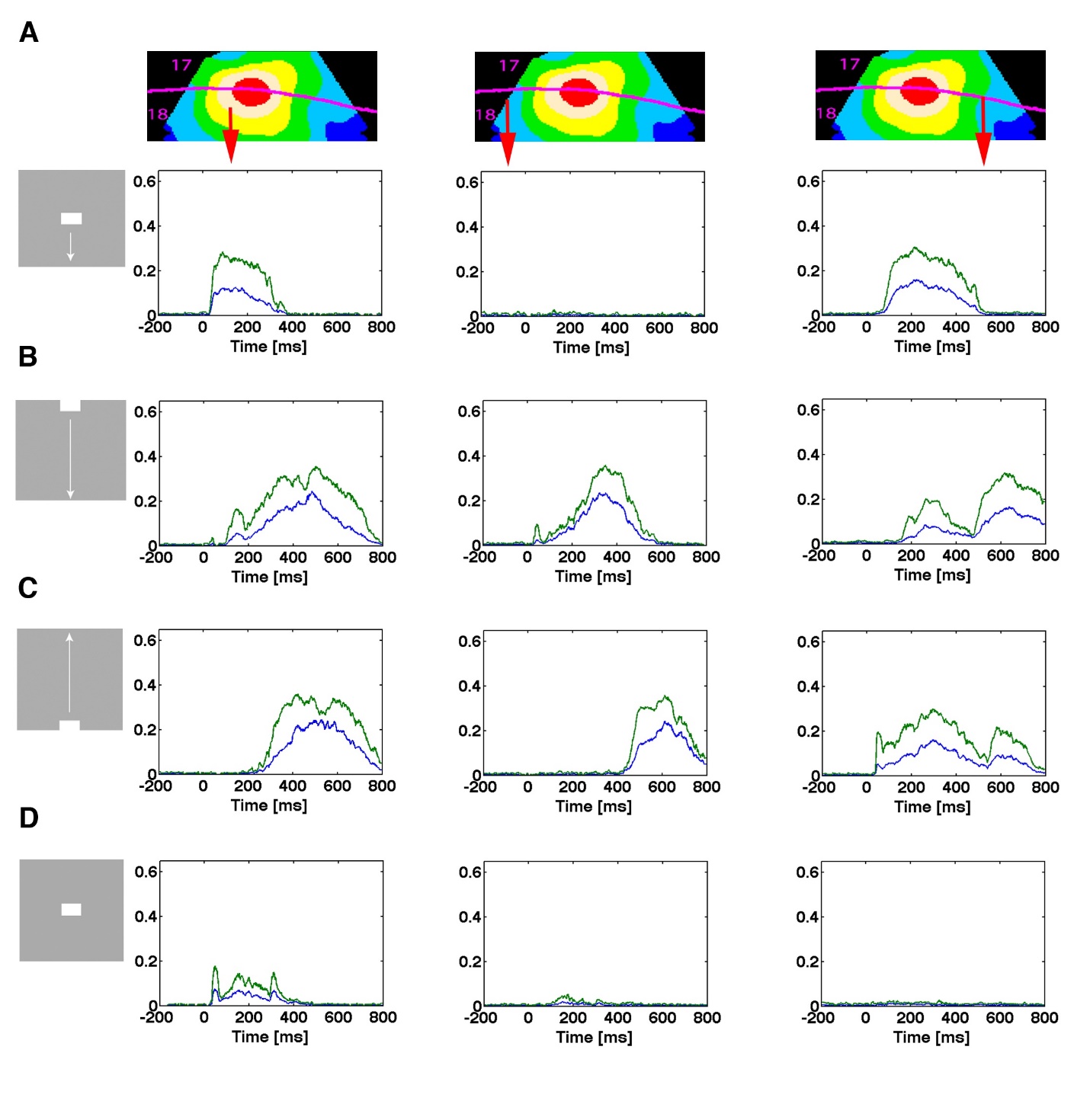


**SFig. 4** *Proportion evoked trials in edge (D) and peripheral vision zones (A and B) of Fig. 3.*

*
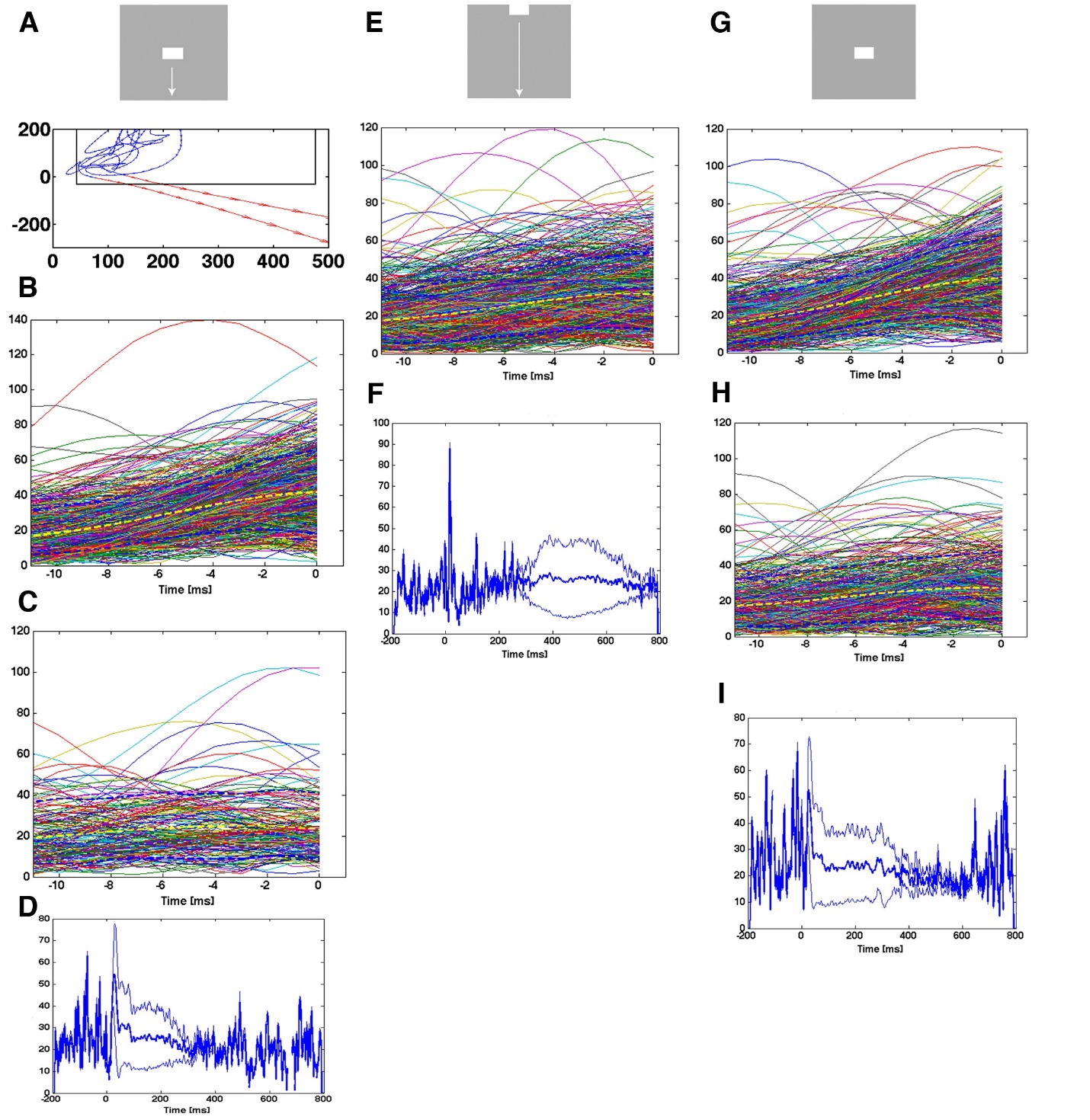
*

**SFig. 5** *Vector flow transition and trajectory speeds*

**A** Single trial vector flow transition from spontaneous ongoing chaotic-like to fast smooth outward directed flow. **B** Acceleration of trajectory speeds prior to the first entry into the evoked state after 20 ms post stimulus. All evoked trials from the 17 electrode penetrations mapping the CFOV. Yellow: mean speed; blue stippled lines : 10%-ile and 90%-ile. A few trials were evoked prior to the sharp transient. **C**  Re-entries into the evoked state after 280 ms. **D, F, I** Mean trajectory speeds and square root of variance for all evoked trials in electrode penetrations mapping the CFOV. **E** Smooth transient driven acceleration of trajectory speeds prior to the entry into the evoked state after 280 ms. **G** Acceleration of trajectory speeds prior to the first entry into the evoked state after 20 ms post stimulus. All evoked trials from the 17 electrode penetrations mapping the CFOV. **H** Re-entries into the evoked state after 280 ms. Stimulus is off at 250 ms.


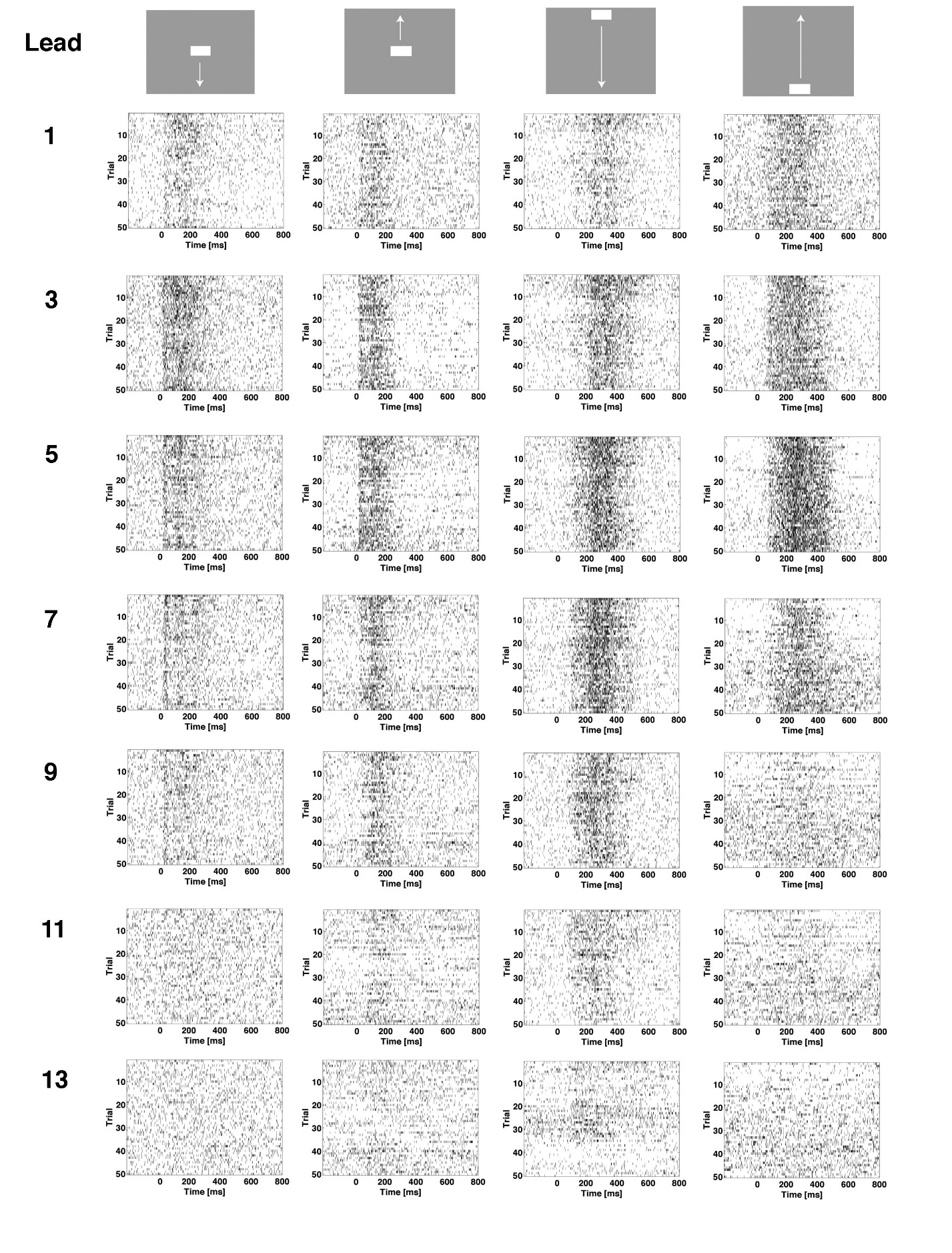


**SFig. 6** *Spike times of single trials.* The raw data raster plots show all trials of the same electrode penetrations of the four scenes of moving objects depicted in Fig. 8 A,B,C,and D . The leads are numbered from the cortical surface,

**Please find the links to the Movies below**

**Movie 1** *50 single trial trajectories in state space in response to a bar moving down from CFOV*. Several projections of state space shown. The projections below the diagonal are redundant. The diagonal show the projections of the single trials for the first 4 principal components. The total variance accounted for by the two principal components is shown on the top of each state space projection in 2 dimensions. The trajectory of a single trial is represented by a red dot shoving the instantaneous position in state space. The dot has a tail showing where the trajectory was up to 20 ms prior to actual time. The yellow dot is the instantaneous center of gravity. Animal 2, penetration 1 in the cortex mapping the CFOV (center of field of view).

**Movie 2** *Smooth entry into the evoked state. 50 single trial trajectories in response to a bar moving down from peripheral FOV* and thus being mapped by the neurons sampled by this electrode after 470 ms. Several trials visit the state space close to the fixed point at (0,0), but quickly leave this zone again. Animal 4, penetration 3 mapping the CFOV. Single trials shown as in Movie 1.

**Movie 3** 50 single trial trajectories. Stimulus: a bar moving up from the CFOV. The electrode was placed in cortex mapping CFOV. The 4 projections of state space by principal components 1 to 8. Note the diminished part of state space exploited in projections PC3,PC4, PC5,PC6 and PC7,PC8. Occasionally a singe trial escapes the attraction of the fixed point in the pre-stimulus period and after the initial drive by the stimulus. Several trials show re-entries into evoked state space.

**Movie 4**  50 single trial trajectories. Stimulus: a bar moving up from peripheral field of view of view. The electrode was placed in cortex mapping CFOV.

**Movie 5**  50 single trial trajectories. Stimulus: a stationary bar presented in CFOV for 250 ms. The electrode was placed in cortex mapping CFOV.
